# Maximum likelihood pandemic-scale phylogenetics

**DOI:** 10.1101/2022.03.22.485312

**Published:** 2022-03-22

**Authors:** Nicola De Maio, Prabhav Kalaghatgi, Yatish Turakhia, Russell Corbett-Detig, Bui Quang Minh, Nick Goldman

**Affiliations:** 1 European Molecular Biology Laboratory, European Bioinformatics Institute, Wellcome Genome Campus, Hinxton, Cambridgeshire, CB10 1SD, UK; 2 Max Planck Institute for Molecular Genetics, Ihnestraße 63-73 14195 Berlin, Germany; 3 Department of Electrical and Computer Engineering, University of California San Diego, San Diego, CA 92093, USA; 4 Department of Biomolecular Engineering, University of California Santa Cruz, Santa Cruz, CA 95064, USA; 5 Genomics Institute, University of California Santa Cruz, Santa Cruz, CA 95064, USA; 6 School of Computing, College of Engineering and Computer Science, Australian National University, Canberra, ACT 2600, Australia.

## Abstract

Genomic data plays an essential role in the study of transmissible disease, as exemplified by its current use in identifying and tracking the spread of novel SARS-CoV-2 variants. However, with the increase in size of genomic epidemiological datasets, their phylogenetic analyses become increasingly impractical due to high computational demand. In particular, while maximum likelihood methods are go-to tools for phylogenetic inference, the scale of datasets from the ongoing pandemic has made apparent the urgent need for more computationally efficient approaches. Here we propose a new likelihood-based phylogenetic framework that greatly reduces both the memory and time demand of popular maximum likelihood approaches when analysing many closely related genomes, as in the scenario of SARS-CoV-2 genome data and more generally throughout genomic epidemiology. To achieve this, we rewrite the classical Felsenstein pruning algorithm so that we can infer phylogenetic trees on at least 10 times larger datasets with higher accuracy than existing maximum likelihood methods. Our algorithms provide a powerful framework for maximum-likelihood genomic epidemiology and could facilitate similarly groundbreaking applications in Bayesian phylogenomic analyses as well.

## 2 Introduction

Genomic data play a crucial role in epidemiology, as exemplified during the SARS-CoV-2 pandemic. Genomic data can be used to track and reconstruct the spread of disease within communities and within and between countries [17, 33, 36, 58], understand the dynamics of transmission [36, 41, 52], estimate the efficacy of containment measures [8, 18, 26, 31], predict future epidemiological dynamics, [57, 58], and for the tracking of pathogen evolution, as showcased by the identification of new SARS-CoV-2 variants and mutations of concern [30, 40].

Investigations of genomic epidemiological data are predominantly based on phylogenetic methods, but analyses of SARS-CoV-2 genome sequence data with existing phylogenetic approaches are becoming more difficult due to the excessive computational resources required by current global datasets consisting of millions of genomes [25]. For example, the daily update of a global SARS-CoV-2 phylogenetic tree is particularly useful in tracking transmission within and between regions and in monitoring new variants [35]. However, performing this task with established phylogenetic software like RAxML [47] or IQ-TREE [39] would require years for each tree update (if possible at all due to memory demand). This is one of the main reasons why tools for tracking viral genome evolution and spread, such as NextStrain [24], and many other genomic analyses, often downsample global SARS-CoV-2 datasets to a few thousand genomes, leading to loss of power and resolution (see e.g. [43, 64]).

Here, we describe MAPLE (“MAximum Parsimonious Likelihood Estimation”), an efficient and accurate maximum likelihood approach for the phylogenetic analysis of large numbers of closely related genomes, as is typical in genomic epidemiology. MAPLE retains many features of typical maximum likelihood phylogenetic methods (e.g., [39, 42, 47]) such as maximum likelihood inference and the use of explicit probabilistic models of sequence evolution, and it combines them with features of maximum parsimony methods (e.g., [56]) that allow it to greatly reduce its computer memory and time demand. To do this, we rewrite the classic Felsenstein pruning algorithm [19] to achieve higher performance for the analysis of genomic epidemiological datasets. Using extensive and efficient tree searches, MAPLE can estimate more accurate maximum likelihood trees than existing alternative approaches, at only a fraction of their memory and time demand, offering for example more than two orders of magnitude speed-up over RAxML and IQ-TREE for large SARS-COV-2 trees.

## 3 Results

### 3.1 New approaches for pandemic-scale likelihood-based phylogenetics

We present here a brief summary of our methods, highlighting five areas in which we have made improvements relative to existing approaches. More detailed descriptions are given in the Methods section.

#### 3.1.1 Concise genome data representation

Genome sequence alignments for genomic epidemiology are often stored in Fasta or other similar format [37]. This means that, despite the fact that genomes within an epidemic are usually extremely similar to each other, all the genome sequences are included in full in the alignment file, requiring one character for each position of each sequence. For large datasets, this represents a substantial memory and computing cost for storage, reading and processing. While it is possible to reduce the size of these datasets using standard compression techniques [45], this only represents a partial solution since the sequences would still need to be uncompressed before analysis.

**Figure 1:**
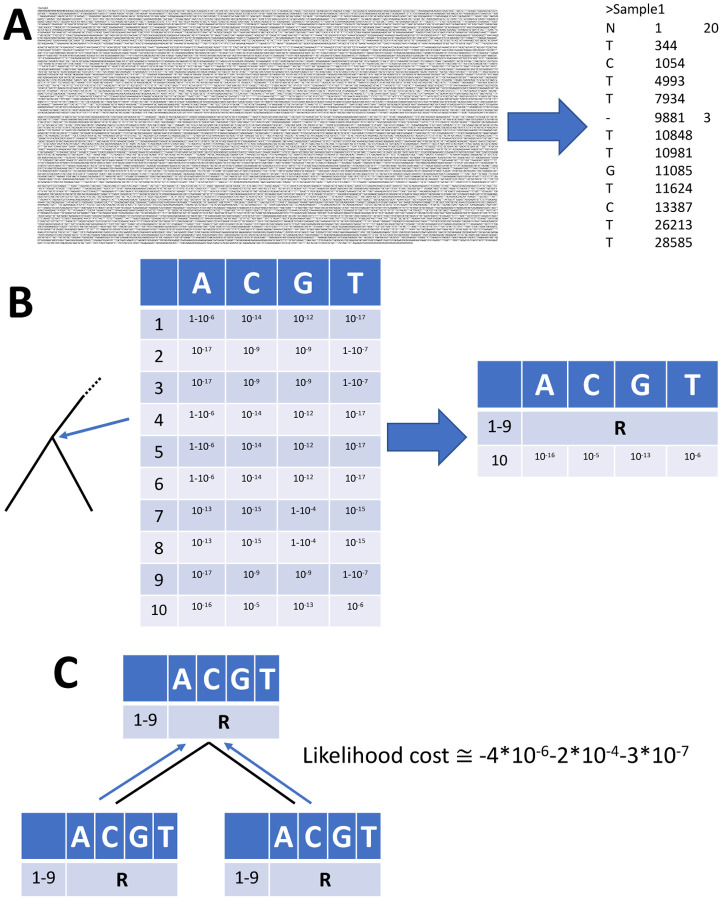
Graphical summary of sequence and likelihood representation and processing. **A** Left: Fasta representation of an individual SARS-CoV-2 genome, which consists of sample name followed by the entire genome sequence. Right: MAPLE format representation, where we record only the differences between the genome under consideration and a reference; the first column in our format represents the character observed, the second column the position along the genome, and the third column (when present) the number of consecutive positions for which the character is observed. **B** Left: an example likelihood vector at an internal node of a phylogenetic tree (shown by the narrow blue arrow; only a small portion of the tree is shown); here for simplicity we show only 10 genome positions. At each position (rows 1–10), each column contains the likelihood for a specific nucleotide. For rows 1–9 the likelihood is concentrated at only one nucleotide, while for position 10 we show an example with more uncertainty. Right: Our representation of the node likelihoods. Assuming that the reference sequence at the first 9 positions matches the most likely nucleotides in the vector (ATTAAAGGT) then for positions 1–9 the likelihood of non-reference nucleotides is negligible and we represent the likelihoods with a single symbol (R) and with the position of the start of the stretch (1). At position 10, due to non-negligible uncertainty, we explicitly calculate and store the four likelihood values. **C**: If for a region of the genome two child nodes are in state R, then their parent is also assumed to be so, and the likelihood contribution of no mutation happening on this stretch on the considered branches is approximated in constant time.

Instead, we represent each genome in terms of differences with respect to a reference genome (Figure 1A and also [15]). This way, we reduce file size approximately 100-fold compared to Fasta files (Figure S1); for example, we reduced the size of the 31-03-2021 global SARS-COV-2 genome alignment from GISAID from 27.84 GB to 224.6 MB (a 124× reduction). Our alignment format (which we call “MAPLE format”) is also substantially more concise than VCF format [9] (which only records entries of non-constant columns of the multiple sequence alignment) when considering many sequences (Figure S1). See Section 5.1 for more details.

#### 3.1.2 Concise phylogenetic likelihood representation

During phylogenetic estimation, likelihood-based approaches calculate likelihoods conditional on ancestral states (in our case we consider nucleotides for simplicity). A likelihood vector at one node of the tree typically contains one entry for each variable position of the genome, with each entry containing four likelihood values (one for each nucleotide, see Section 5.2 and [22]). However, in genomic epidemiology, due to the similarity of the genomes considered, likelihoods are most often highly concentrated at only one of the four nucleotides. To exploit this feature, we approximate the likelihoods and represent them concisely; when the likelihood is highly concentrated (based on a threshold) in the reference nucleotide at each site of a stretch of the genome, we simply record the stretch as coinciding with the reference (Figure 1B). At sites at which the likelihood is not concentrated at one nucleotide, we keep track of all the four nucleotide likelihoods. See Section 5.2 for more details. This approach both saves memory and permits time savings through reducing the complexity of likelihood calculations, as described next.

#### 3.1.3 Efficient likelihood calculation

While our concise likelihood representation provides memory efficiency, we also present a time-efficient approach for calculating these likelihoods that similarly reduces the computational demand of likelihood-based phylogenetics with many similar genomes. The main principle of our algorithm is that we can efficiently approximate the likelihood contribution at a node of the tree for a long region of the genome if, at this node and in this region, the only likely evolutionary history is the one in which the reference nucleotides have been inherited without mutating (Figure 1C). We consider the scenario of short evolutionary distances, which is typically the case for SARS-CoV-2 and will likely be true for future epidemics with high rates of sampling of pathogen genomes. In this case, if the same nucleotide is observed at a given site for the two children of a node, then it is extremely unlikely that the same nucleotide was not also at the node and site considered; as an approximation, we therefore do not keep track of these unlikely mutational histories. For this reason MAPLE can be considered a hybrid combining the efficiency of parsimony-based phylogenetic methods (in particular UShER [56]) with the potential accuracy and applicability of likelihood-based ones. For more details on our likelihood calculation algorithm, approximations and assumptions, see Section 5.3.

#### 3.1.4 Efficient tree extension

**Figure 2:**
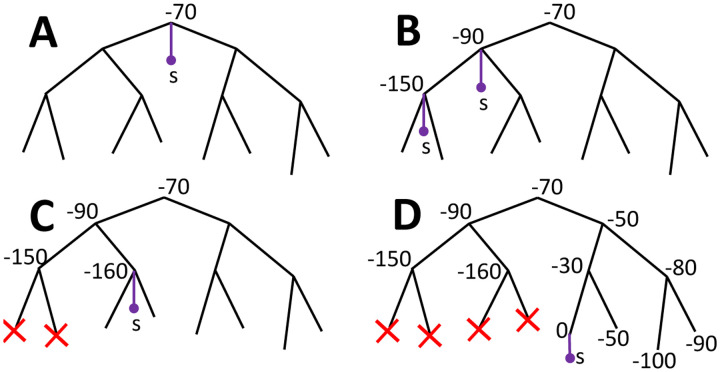
Graphical summary of phylogenetic placement approach. **A** To search for the best placement of a new sample *s* (here represented by a purple dot and branch) on the current tree, we first attempt the placement at the root, which in this case results in a relative log-likelihood score of −70. **B** We iteratively visit descendant nodes by preorder traversal and for each visited node we attempt placement (in reality we also attempt placement onto branches, not only nodes). **C** When the log-likelihood score decreases two times consecutively and falls below a certain threshold relative to the best placement found so far, we do not visit further nodes downstream (here crossed in red). **D** The placement with the highest score at the end of this process (in this case with cost 0) is selected for the addition of *s* to the tree.

Our phylogenetic inference algorithm consists of two stages. In the first stage, starting from a tree containing only one sample, we add one sample at the time onto the current tree by maximum likelihood (stepwise addition: SA, [53]). In order to place a sample on the tree, we first evaluate the placement at the root, and then compare this log-likelihood score against those for the placements at its children. In our novel SA strategy, the search for the best placement location proceeds by pursuing these calculations into high-scoring regions of the tree and avoiding placement evaluations in unlikely tree regions (Figure 2). This greatly reduces the computational demand of our approach at a potential small cost in accuracy. For more details, see Section 5.5. The SA stage provides a full starting tree that we attempt to further improve as described in the next section.

#### 3.1.5 Efficient tree space exploration

After we obtain an initial tree by iteratively placing all of the samples, we proceed to the refinement stage where we optimize the tree topology. To do this, we use subtree pruning and regrafting (SPR, [53]) moves, where we sever a subtree from the tree and attempt to attach it somewhere else on the tree. Similar to our SA procedure, we traverse the tree (this time starting from the current location of the subtree) to look for the best re-attachment. Again, we improve on typical SPR implementations by using log-likelihood thresholds to avoid unpromising regions of the tree. By storing and re-using likelihoods at tree nodes, and by avoiding likelihood updates of nodes whose likelihoods are not affected by a changes in the tree, we can also perform each SPR move very efficiently. For more details, see Section 5.6.

### 3.2 Accuracy and computational demand of tree estimation

**Figure 3:**
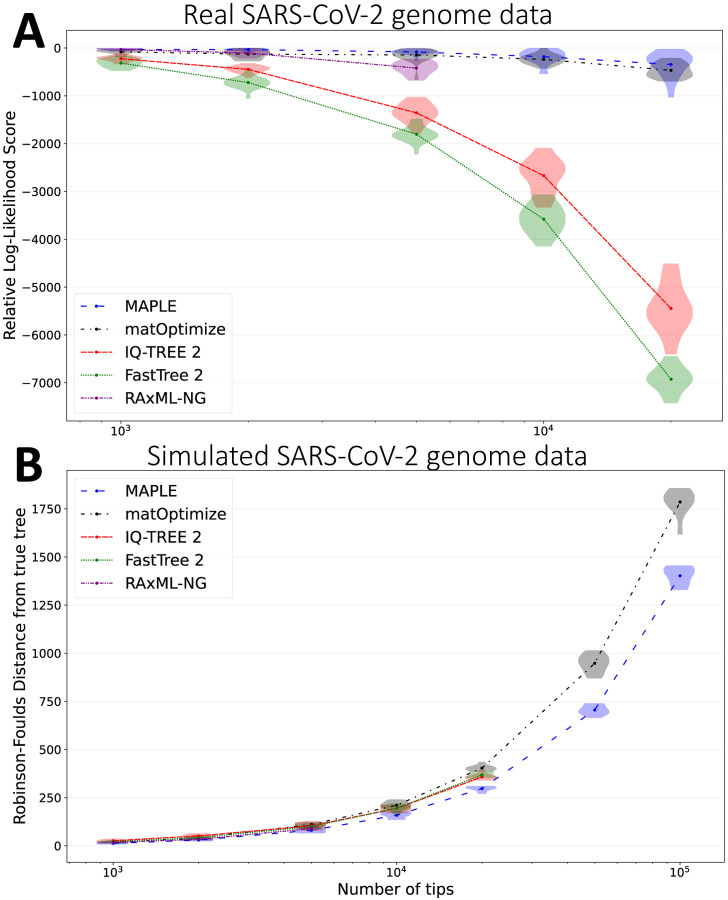
Comparison of accuracy of different methods for phylogenetic inference from SARS-CoV-2 genomes. MAPLE consistently delivers higher accuracy in SARS-CoV-2 phylogenetic estimation. Each phylogenetic inference method considered is represented by a different color and line style (see legends within the plots). Values on the X axes show the number of samples included in each replicate. We ran each method up to the dataset size that could be analysed with our computational resources due to time and memory limitations. Each violin plot summarizes values for 20 replicates, and dots represent mean values. **A** Relative log-likelihoods of phylogenies inferred by different methods on real SARS-CoV-2 data; for each replicate, and for each method, we plot the difference in log-likelihood score between the tree inferred by that method, versus the highest log-likelihood score obtained by any method for that replicate; so, for each replicate, at least one method has a relative log-likelihood score of exactly 0. Higher values on the Y axis represent more likely estimates. We could only run log-likelihood comparisons up to datasets of 20,000 samples due to the computational demand of log-likelihood evaluation (see section 5.11 for details on log-likelihoods evaluation). **B** Robinson-Foulds distances between estimated trees and true trees in simulated data (the “basic simulation scenario” described in Section 5.10). Higher values correspond to more errors in phylogenetic estimation.

MAPLE appears consistently more accurate than existing large-scale phylogenetic inference methods (we considered IQ-TREE 2 [39], FastTree 2 [42], RAxML-NG [28], RAxML [47], UShER [56] and matOptimize [63]), both on real and simulated data (Figures 3, S2 and S3); when inferring large phylogenies (e.g. relating 100,000 samples), MAPLE typically prevents hundreds of phylogenetic estimation errors.

The methods used for accuracy estimation are described in Section 5.11. Real datasets used for these comparisons are described in Section 5.9, while simulations are described in Section 5.9; briefly, we simulated SARS-CoV-2 genome evolution with or without rate variation across the genome, and with or without sequences ambiguities typically observed in SARS-CoV-2 genome data.

The higher accuracy of MAPLE is likely the result of its more extensive phylogenetic tree search compared to the other maximum likelihood methods considered here (IQ-TREE 2, FastTree 2, RAxML-NG and RAxML), which is enabled by the reduced computational demand of our data and likelihood representation and processing algorithms. matOptimize, a new maximum parsimony approach tailored for phylogenetic inference from large SARS-CoV-2 datasets, also performs a similarly extensive tree search; in this case, the higher accuracy of MAPLE is likely the result of its explicit probabilistic modeling of sequence evolution.

**Figure 4:**
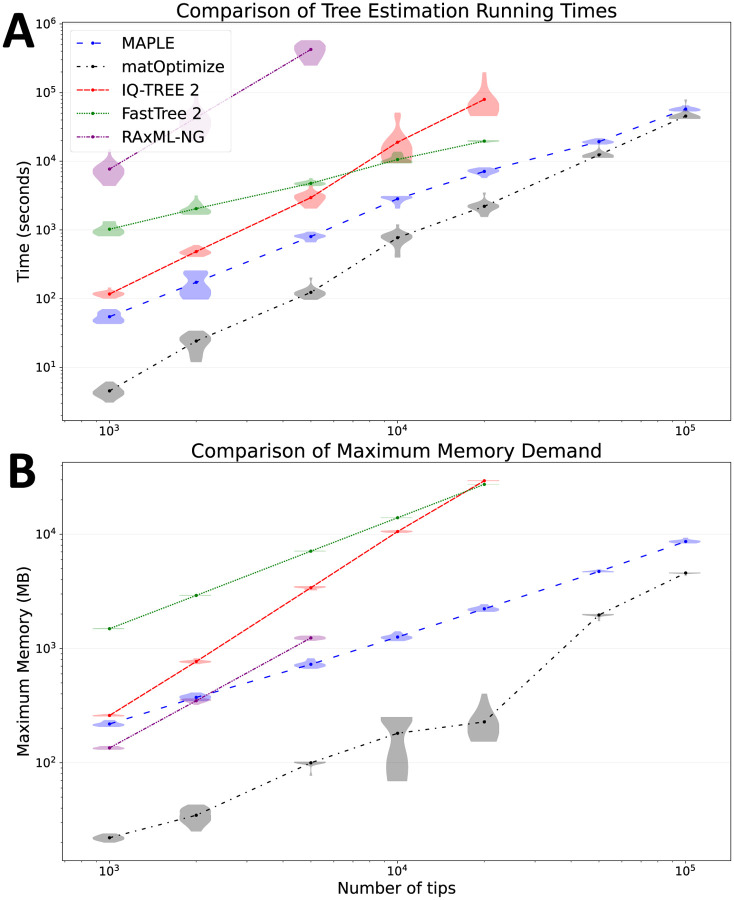
Comparison of running times and memory demand of different methods for phylogenetic inference from real SARS-CoV-2 genomes. MAPLE allows estimation of larger trees than current maximum likelihood methods. **A** seconds required to run each method considered. **B** maximum RAM memory demand in MB required to run each method considered. In both cases on the X axis is the number of sequences in each replicate. All axis scales are logarithmic. We ran each method up to the dataset size that could be analysed with our computational resources due to time and memory limitations. Each violin plot summarizes values for 20 replicates, and dots represent mean values.

Despite its more extensive and accurate tree search, MAPLE also typically provides a substantial reduction in computational demand compared to the other maximum likelihood methods considered here, requiring about 100-fold less runtime than RAxML-NG (Figures 4A and S4), the next most consistently accurate approach. The memory demand of MAPLE is about 10 times lower than FastTree 2, and is less than those of RAxML-NG or IQ-TREE 2 for trees with more than 2,000 sequences, with MAPLE becoming relatively more memory efficient as larger trees are considered (Figures 4B, S4–S6). With our available computational resources, it was possible to run MAPLE on datasets 5 times larger than those allowed by IQ-TREE 2 and FastTree 2, and 20 times larger than those allowed by RAxML-NG, showing that, in addition to estimating more accurate SARS-CoV-2 phylogenies, MAPLE also allows phylogenetic inference on larger datasets than existing maximum likelihood approaches.

MAPLE requires a runtime similar to the parsimony-based matOptimize for the largest tree considered here (relating 100,000 samples). matOptimize shows lower memory footprint than MAPLE in the sizes of trees considered here, but its memory demand approaches that of MAPLE as tree size increases.

## 4 Discussion

By rewriting the classical Felsenstein pruning algorithm, by including features of parsimony-based phylogenetic inference in a likelihood-based context, by using efficient approximations, and by using more concise data representation, we can achieve substantial reductions in memory and time demand and increase in accuracy compared to popular maximum likelihood approaches when inferring SARS-CoV-2 phylogenies. On large phylogenies (e.g. including 100,000 samples) MAPLE can prevent hundreds of phylogenetic inference errors. Also, with the same computational resources, we were able to estimate phylogenies at least 5 times larger than those allowed by IQ-TREE 2 and FastTree 2, and 20 times larger than those allowed by RAxML-NG. We anticipate that this increase in efficiency may even improve for larger datasets with millions of samples.

Beyond SARS-CoV-2, our approach will be equally useful in any scenario with many sequences and with short evolutionary distances, such as most scenarios in genomic epidemiology. This includes genomic datasets with many samples from an individual pathogen, including for example large collections of *M*. *tuberculosis* genomes (e.g. [5]) or influenza genomes (e.g. [46]), and collections of genomic data from possible future pandemics. The computational advantages of MAPLE are expected to increase with genome size, genome incompleteness, and number of sequences considered, and could therefore also benefit phylogenetic analyses from large collections of somatic mutations (e.g. [6]).

The applicability of our methods goes beyond maximum likelihood phylogenetics. The same data structures and algorithm in MAPLE could also be used in a Bayesian setting, since Bayesian phylogenetic methods use the same genetic data (multiple sequence alignments) and the same likelihood calculation algorithms as maximum likelihood phylogenetic methods, and so could benefit from the same reduction in computational demands. For example, the Bayesian phylogenetic package BEAST [3,50] is very frequently used to analyse genomic epidemiological datasets, and we expect that its computational demand could be strongly reduced in these applications using our approaches.

By avoiding matrix exponentiation, and in general long-distance modeling of sequence evolution, MAPLE can easily and efficiently be generalized to complex models of sequence evolution, for example to model non-stationary and highly variable mutation rates, such as those observed in SARS-CoV-2 [14], as well as to model codon evolution [1] and indels [11, 51].

For these reasons, we expect that in the future MAPLE and its algorithms will expand the computational toolkit of genomic epidemiology and could improve our preparedness for combating future epidemics.

## 5 Methods

Our approach to tree inference differs from traditional maximum likelihood phylogenetic approaches in that we concisely represent genetic sequences (see Section 5.1) and partial likelihood vectors (see Section 5.2). We not only make use of these concise representations to reduce memory demand, but we also use novel algorithms to efficiently calculate and update likelihoods (see Section 5.3). To further reduce time complexity in likelihood calculations, and to allow non-stationary substitution models (which better describe SARS-CoV-2 evolution [14]) we adapt the strategy of Boussau and Gouy [4] to our scenario, storing partial likelihoods from different subsets of the data at each node, as described in Section 5.4.

Our first step in phylogenetic inference is to use maximum likelihood Stepwise Addition (SA, [53]) to build an initial tree from scratch, given only the input genetic data. This means that, starting from a tree containing just one sample, we iterative add (or “place”) new samples to the current tree one at the time, so that at each step the tree grows in size by one sample. We aim to do this both efficiently and accurately, so that the initial tree itself already represents a reasonable phylogenetic estimate. In fact, at each step, we place a new sample on the tree so to minimize the likelihood cost of the addition, but at the same time avoiding the traversal of the full phylogeny. At the same time as we build the initial tree, we also estimate the substitution process (the model of sequence evolution). The process of building the initial tree is described in more detail in Section 5.5.

Once we finalize the initial phylogenetic tree by stepwise addition, we then attempt at improving the tree by proposing changes to its topology and branch lengths. We use SPR (Subtree Prune and Regraft) moves to change the tree topology, similar to other phylogenetic methods, but instead of limiting the search radius of the SPR moves in terms of number of branches separating the initial and proposed placement of subtree, as typically done, we instead use an approach based on likelihood thresholds that combines efficiency with accuracy; for more details, see Section 5.6. After a number of series of these tree updates (with number specified by the user), the tree obtained represents the maximum likelihood tree estimate of MAPLE.

Most of the symbols and expressions used throughout the Methods section are summarized in Table 1.

### 5.1 Concise representation of genomic epidemiological sequence data

In genomic epidemiology, and for example in SARS-CoV-2 genomic data analysis, the genomes considered typically differ only slightly from each other, and therefore from a common reference genome. This makes a typical alignment file, such as a Fasta file, particularly redundant, since it contains the full genome of each sample, despite the fact that this is almost the same genome repeated many times.

Alternative approaches exist to represent this type of data concisely, for example through a VCF file or through a mutation-annotated phylogenetic tree (MAT, see e.g. [34, 56]). The VCF format uses a column for each position that is variable in the alignment, that is, at which at least one genome differs from the reference; this makes this format substantially redundant when large numbers of sequences are considered as in global SARS-CoV-2 genome data. MAT formats represent genetic alignment data as mutations along a phylogenetic tree, and they are an extremely efficient way to represent genetic data that resulted from sequence evolution along a tree with short branches. However, MAT formats require the availability of a realistic tree, whose inference is the purpose of our methods; for this reason, here we focus on the representation of alignment data prior to tree inference.

We now describe the efficient, simple, concise and human-readable format that we use for representing an alignment of closely related genome sequences, which we call MAPLE format. Similar to VCF and CRAM [23] files, we express each genome sequence in terms of its differences (substitutions and deletions) with respect to the reference, representing only the differences of each genome compared to the reference. Unlike the MAT format, our approach does not require knowledge of a tree.

In order to allow calculation of phylogenetic likelihoods accurately, in our format we also efficiently record ambiguous positions (typically represented with IUPAC ambiguity characters), and deleted or non-sequenced portions of the genomes (typically represented with gap “-” and “N” characters, respectively).

As an example, assume that a reference genome “Reference” is made of a sequence of 20 “A” characters, that is, it consists of the sequence

>Reference
AAAAAAAAAAAAAAAAAAAA

(here represented in Fasta format). This sequence is of course unrealistically short and is only meant as a simple example. Assume that a sampled genome “Sample” consists of the sequence:

>Sample
NNNNNAAAAA - - - AAAAATA

when aligned to the reference, as would be represented in Fasta format. We instead represent this same information with the format:

>Sample
N 1 5
− 11 3
T 19

where in each entry (row) the first column represents the type of difference with respect to the reference, the second column in each row represents the position (along the reference genome) of the difference, and the number in the third column (which in practice we only require for “N” and “-” entries represents how many consecutive positions have this same character.

In the rest of the paper, we assume that gap “-” characters and missing data “N” characters have the same interpretation, that is, a complete lack of information, as typical in phylogenetics; however, we still represent gaps and unsequenced positions with these distinct characters since other applications might treat these positions differently, for example by modeling indel events within a phylogenetic context.

The benefit of this format becomes clear with larger genomes (e.g., >29,000 bp for SARS-CoV-2 and millions of bases for bacterial genomes) and with sequenced genomes closely related to the reference. For example, we downloaded the 31-03-2021 unmasked Fasta alignment of all full SARS-CoV-2 genomes from GISAID (containing 915,508 sequences), which had a size of 27.84 GB. After representing it in the format above, without any loss of information, the size was reduced to 224.6 MB, i.e. a reduction of about 99.2%, and contained on average about 28.1 entries per sequence. For bacterial datasets we would expect the advantage to be even more evident, due to the larger reference genome. Further comparisons of file sizes for our format compared to Fasta and VCF format (the latter as used in input for UShER) for the alignments considered in our method comparisons are presented in Figure S1. We can see that our format results in files 100 times smaller than Fasta. VCF format is quite efficient for smaller numbers of sequences, but its file size grows faster than other formats with increasing sequence numbers, probably because, as more samples are included, the number of variable positions considered also increases.

Another advantage of the MAPLE format is that, compared to traditional compression methods, one does not need to re-build the original file in order to use it. In the following, we assume that genome data is represented in this format, and we will use a similar idea to efficiently represent partial likelihoods at phylogenetic nodes. This replaces the traditional consideration of sequence alignments as a series of ‘site patterns’, with likelihood calculations performed for each distinct pattern individually (sometimes referred to as ‘aliasing’ or ‘site pattern compression’ [21]).

While we think our format is very convenient for use in genomic epidemiology, we also note that it is extremely similar to formats previously used in bioinformatics, such as the one corresponding to the “–cs” option in minimap2. We do not claim novelty or superiority of our format with respect to these other formats — we simply think it will be important in the future to extend multiple sequence alignment and phylogenetic inference software, among others, to allow these types of formats in addition to the more traditional Fasta and Phylip formats so to more efficiently store and process genomic epidemiological data. MAPLE format is the one we have adopted at present.

In the following we will assume that, as typical for SARS-CoV-2 data, all genomes are individually aligned to a reference, and that therefore insertions are not included in the global alignment. In the future we however plan to extend our methods to more properly model insertion events and make use of insertion data.

### 5.2 Concise representation of ancestral sequences and sequence uncertainty

To reduce memory demand during likelihood calculations, in addition to representing sequence data efficiently (as discussed in the previous section), we also want to reduce time and memory demand for representing and processing ancestral sequences and partial likelihoods at internal nodes of the tree. Here we describe the concise data structure that we use for representing phylogenetic likelihoods; more details on how we calculate and update them are given in the following sections.

The underlying principle is similar to the one in the previous section and in UShER [56]: we want to represent sequence information concisely as a set of differences with respect to the reference. The complication here is that in addition to storing sequences, we also want to store information regarding the uncertainty associated with each nucleotide, as embodied in the partial likelihoods of different nucleotides at different genome positions at internal nodes of the phylogeny [19].

Given a node *n* of the phylogenetic tree *ϕ*, a column *i* of alignment *A* containing site pattern (nucleotides) *A*_*i*_, and an evolutionary model *M*, the partial likelihood at *n* and *i* of nucleotide *X* is typically defined in phylogenetics as:

(1) $$p_{i}^{n}(X)=p\left(A_{i}^{n}|X,M,\phi \right)$$

where $A_{i}^{n}$ are the subset of observations in *A*_*i*_ corresponding to the descendant leaf nodes of *n*. (Typically non-leaf nodes have no corresponding alignment rows, that is, observed genomes.) These partial likelihoods are typically calculated with the Felsenstein pruning algorithm [19]; in total, there are 4 × *L* × |*ϕ*| such likelihoods that need to be computed, stored and updated during phylogenetic inference, where *L* is genome length and |*ϕ*| is the number of nodes in *ϕ*. Due to genome size (for SARS-CoV-2, > 29,000 bp, but for bacteria typically millions of alignment positions are included in genome-wide alignments) and number of nodes in the tree (which can be millions for SARS-CoV-2 and other genomic epidemiological datasets), calculating and storing partial likelihoods can be a limiting factor in terms to time and memory demand in maximum likelihood and Bayesian phylogenetic inference. A typical way to reduce this cost is to collapse identical alignment columns and simply keep track of how many columns contain each unique pattern. However, as the number of samples increases, the number of alignment columns exhibiting the same pattern typically decreases, making this shortcut insufficient to address the limitations of classical phylogenetic likelihood algorithms.

To address this issue, we replace partial likelihood vectors with more concise structures that we call “genome lists”. Each entry of a genome list represents phylogenetic partial likelihoods for either one position of the genome or for a set of consecutive positions that share similar features — similarly to our alignment format. An important difference from the traditional Felsenstein pruning method is that, for each genome position and tree node, we only keep track of relative partial likelihoods among the four nucleotides, and not exactly of each $p_{i}^{n}(X)$; in other words, we aim at tracking values ${\tilde{p}}_{i}^{n}(X)=p_{i}^{n}(X)/\sum_{D}p_{i}^{n}(D)$. The advantage of this feature is that changes in the tree (such as the addition of a new sample or a change to the current tree topology or the length of a branch) typically affect only the relative likelihoods of the nodes in the phylogenetic vicinity of modified parts of the tree, and so tree space exploration can be performed very efficiently. Below, we explain how global likelihoods can still be evaluated with this approach.

An entry of our genome list is a tuple of four elements (*T*, *i*, *l*, *v*), comprising:

- an entry “type” *τ*; the permitted types are “**R**”, to represent collections of contiguous sites that are identical to the reference, that is, sites where the partial likelihoods are all concentrated at the reference nucleotide; type “**N**” to represent contiguous sites that contain no descendant sequence information, that is, sites where all four nucleotides have the same partial likelihoods; type “**A**”, “**C**”, “**G**” and “**T**” to represent individual sites where the corresponding non-reference nucleotide is the ancestral one at the node with negligible uncertainty, that is, the partial likelihood mass is all concentrated in one non-reference nucleotide; and type “**O**” (“other”) to represent positions where multiple nucleotides have non-negligible relative partial likelihoods.
- a “position” *i* representing the position of the reference to which the entry refers. If the entry represents a stretch of sites, this element is the position of the first one (from 5′ to 3′) of these sites. The last position of the entry need not be specified explicitly since it is the one just before the position element of the next entry, unless the entry is the last one.
- the “branch length” *l* represents the evolutionary distance (using the same unit used to represent branch lengths) between the considered node *n* and the location in the tree where the partial likelihoods contained or represented by the genome list entry refer to. *l* is used to pass likelihood information between nodes of the phylogeny without having to update or recalculate them, which is useful to retain accuracy without compromising computational demand. The rationale behind this entry element will become more clear in the next Section.
- relative partial likelihoods (“partials”) *v*, representing the vector ${\tilde{p}}_{i}^{n}(X)$ for the position considered — only needed for entries of type “**O**”.

The reason for having type “**R**” in addition to types “**A**”, “**C**”, “**G**” and “**T**” (which could also be used to represent a position identical to the reference) is that the “**R**” type allows us to represent and process stretches of the genome that are identical to the reference in a computationally efficiently way, as will be explained below.

Where we have made use of the concept of negligibility to distinguish entries of type “**O**” from the others, in practice we define negligibility through an arbitrary threshold *ϵ* with default value *ϵ* = 10^−7^, that is, a site is of type “**O**” only if at least two nucleotides have a relative partial likelihood ${\tilde{p}}_{i}^{n}(X)>\epsilon$.

As an example, we can consider the sample in the previous section

>Sample
N 1 5
− 11 3
T 19

and the same reference genome comprising 20 “A” nucleotides. Under these assumptions, at the terminal node of the phylogeny corresponding to “Sample”, we have the genome list

{[N,1],[R,6,0.0],[N,11],[R,14,0.0],[T,19,0.0],[R,20,0.0]};

We ignore branch length elements (third elements in each entry) of entries of type “**N**” since they are redundant, and similarly for the “partials” elements (fourth element in each entry) of all entries above.

In bioinformatics, IUPAC nucleotide ambiguity codes are used to represent positions of a sequence where a nucleotide is not known with certainty [7]. For example, character “Y” represents a position where either nucleotide “C” or “T” might be present. If instead of a “T” character at position 19 we observed an ambiguity code “Y”, then the corresponding genome list entry would have been

[O,19,0.0,(0.0,0.5,0.0,0.5)].

These relative partial likelihoods are those typically used in phylogenetic inference for ambiguity characters (although usually they are not normalised). In the future, other implementations might be possible to account for the fact that ambiguity codes in genomic epidemiology might represent the presence of within-host variants (see e.g., [16]) or sequencing errors [13, 29, 55] rather than general uncertainty.

For now, branch length elements of the entries of the genome list do not appear useful, as they have been all 0. These branch length elements represent the evolutionary distance (in the same units used for branch lengths in the tree *ϕ*) between the node to which the genome list refers, and the location in the tree for which the partial likelihoods represented in the element were calculated. For the tips of the phylogenetic trees, the branch length elements will always be 0 since the genome lists and likelihoods are initialized at each tip. However, branch length elements *l* allow us to “pass” partial likelihood information from children nodes to parent nodes without having to perform recalculations or changing the structure of the genome list elements, which could be computationally demanding. Consider the example of an internal node *n* with descendants Sample (as above) and Sample2 separated from *n* respectively by (short) branches of length *l*_1_ and *l*_2_ (in units of time or expected substitutions per site), and assume Sample2 has data

>Sample2
N 19 3

While the general algorithms for calculating genome lists will be presented in detail in the following sections, for now, to exemplify the use of branch length elements, we describe the genome list for *n*, which is:

$$\left\{\left[R,1,l_{2},\right],[R,6,0.0,],\left[R,11,l_{2},\right],[R,14,0.0,],\left[T,19,l_{1},\right],\left[R,20,l_{1},\right]\right\}.$$

As can be seen here, branch length elements can take a range of values, here 0.0, *l*_1_ and *l*_2_ and at nodes further up the tree *ϕ* many more values can become possible. These values play an integral part to the partial likelihood calculations described in the following sections; as shown here, they are useful for avoiding costly likelihood calculations when one (and only one) of the two children of an internal node has type “**N**” at a site. In this case, parent node *n* can use the same genome list type (for example “**T**” at position 19) as the child node, despite the fact that nucleotide T is not observed at node *n* but at its child Sample. In this case, branch length *l*_1_ is used to record the distance between *n* and the point in the tree where T was actually observed (the tip corresponding to Sample).

**Figure 5:**
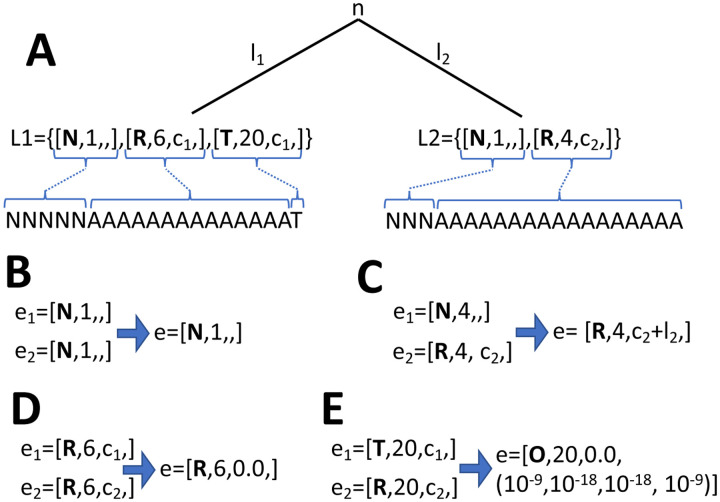
Graphical example of the merging of genome lists. Here we consider the example genomes considered in the text, with a reference genome consisting of 20 “A” nucleotides, partial likelihood genome lists *L*_1_ and *L*_2_, and a phylogeny with two branches, one of length *l*_1_ leading to Sample1 and genome list *L*_1_, and one of length *l*_2_ leading to Sample2 and genome list *L*_2_. **A** Graphical representation of the phylogeny, of the two observed genomes and of the partial likelihood genome lists at the tips. Blue parentheses and lines highlight the correspondence between genome list entries and portions of the observed genomes at the tips of the tree. Our aim here is to calculate the partial likelihood genome list for the most recent common ancestor *n* of Sample1 and Sample2. Parameters *c*_1_ and *c*_2_ here usually would have value 0 since they refer to tips of the tree - however, for internal nodes these values could be strictly positive, so we use general parameter names here to give more generality. **B** For the first intersection fragment of the genome consisting of the first three positions, both children node partials contain no information (they are of type **N**), so the same is true for their parent (which will also be of type **N**). **C** For positions 4 and 5, Sample1 provides no information while Sample2 presents the reference allele. In this case the parent node genome list entry will be of type **R**, but we also keep track of the branch length *l*_2_ using the branch length element of the entry. **D** From positions 6 to 19 both child node genome list entries are of type **R**, and so the same is true for the parent node at their intersection fragment. The corresponding parent node genome list entry will have branch length element set to 0, which is the same as considering the reference alleles observed exactly at the parent node. **E** At the last position of the genome, while at Sample1 we observe nucleotide “T”, at Sample2 we observe nucleotide reference “A”; in this case, the parent node genome list entry will be of type **O**, and we calculate an explicit partial likelihood vector with the relative likelihoods of all four nucleotides. The branch length element of the genome list entry is set to 0, since the relative partial likelihood refer to the parent node.

A full description of how genome lists are created and processed is given in the next section, with a graphical example in Figure 5. A genome list contains all necessary information for us to define our likelihood calculation algorithms presented below, and thus replaces the full vector of partial likelihoods typically employed in phylogenetics for the Felsenstein pruning algorithm.

Note that the choice of reference genome can have a non-negligible effect on the computational efficiency of our formats and our algorithm. Ideally, a reference genome should be as close as possible to the sampled sequences considered, and using the consensus sequence of the while alignment is a reasonable choice. In the future it could make sense to adopt different reference genomes for different blocks of the phylogenetic tree.

### 5.3 Fast pruning algorithm in the limit of short branch lengths

In this section we describe the approach we use to calculate phylogenetic likelihoods, based on the data structure described in the previous section 5.2. Instead of calculating likelihoods one site at the time, as in the classical Felsenstein pruning algorithm [19], we use an approach specific for phylogenetic trees with short branches. We first describe our assumptions regarding the sequence evolution model (section 5.3.1), which is essential for calculating phylogenetic likelihood, and then we describe our algorithm for calculating these likelihoods (section 5.3.2). Throughout this section we assume that we are given a phylogenetic tree *ϕ*; in further sections below we describe how this likelihood calculation algorithm is actually used to infer *ϕ*.

#### 5.3.1 Sequence evolution model

As is standard in phylogenetics, as assume that sequence evolution is a continuous-time and finite-space homogeneous Markov process, where all sites evolve independently [22]. For simplicity we assume nucleotide sequences, and we assume a nucleotide substitution process determined by a substitution rate matrix *Q* whose entries *q*_*XY*_, for any *X* ≠ *Y*, represent instantaneous rates of substitution from nucleotide *X* to nucleotide *Y*.

Diagonal entries *q*_*XX*_ are conventionally defined such that the sum of the values of each row in *Q* is 0; this allows the use of matrix exponentiation to calculate transition probability matrices in typical maximum likelihood phylogenetic methods. Instead, here we use first order approximations over branch lengths, as we assume that the latter are always short:

(2) $$P(Y|X,l)\approx lq_{XY}$$

when *X* ≠ *Y*, where *P*(*Y* |*X*, *l*) is the probability of nucleotide *X* evolving into nucleotide *Y* after divergence distance *l*. Similarly:

(3) $$P(X|X,l)\approx 1+lq_{XX}.$$

Using these approximations brings substantial computational advantages compared to classical approaches based on matrix exponentiation and matrix-vector multiplications. It also has the further benefit of increased numerical stability, which allows us to use non-stationary non-reversible substitution models such as UNREST [61].

#### 5.3.2 Efficient calculation of likelihoods

Here we describe our algorithm to efficiently calculate the partial likelihoods at a phylogenetic node *n*. Similarly to the Felsenstein pruning algorithm, we assume that we have already calculated the same likelihoods for the children nodes of *n*, if *n* is not a tip of the tree. For simplicity, we assume that the tree *ϕ* is binary and rooted, that is, each internal node has exactly two children. Multifurcations can still be represented, using branches of length 0. While here we assume that a generic tree *ϕ* with branch lengths is given, in further sections below we describe how an initial tree is inferred and updated. Given that our approach allows numerically stable phylogenetic inference with non-stationary models, the root of the phylogeny can be estimated with sufficient accuracy when the substitution process is sufficiently non-stationary [59]. Otherwise, the rooting of the tree can be assumed to be arbitrary.

Instead of calculating full partial likelihood vectors with likelihood values for each alignment column and nucleotide, as in traditional approaches, we estimate the equivalent, but more concise, genome list described in section 5.2. Our genome lists keep track of relative likelihood values, and the total likelihood component not accounted for in these normalized relative likelihoods is tracked with the “total likelihood” parameter *K*, whose exact use we explain below.

We have already shown in section 5.2 how to initialize genome lists for terminal nodes of the tree. Now, we assume that *n* has children nodes *b*_1_ and *b*_2_ with genome lists respectively *L*_1_ and *L*_2_. We also assume that *b*_1_ and *b*_2_ are separated from *n* by branches of length *l*_1_ and *l*_2_. We want to calculate the genome list *L*_*n*_ of node *n*, which we obtain by “merging” information from *L*_1_ and *L*_2_.

When needed, for example to estimate the total likelihood of the tree, the total likelihood *K* of *n* is initialized to *K* = *K*_1_ + *K*_2_, where *K*_1_ and *K*_2_ are the total likelihood values for *b*_1_ and *b*_2_. However, in most circumstances, for example when we only need to calculate the additional likelihood cost of adding one sample to an existing tree, then we are only interested in relative likelihoods and we initialize *K* = 0.

Given the two genome lists *L*_1_ and *L*_2_, we split the genome into segments, where each segment corresponds to genome positions that all belong to the same genome list entry in *L*_1_, and also all belong to the same entry in *L*_2_. More formally, assume that entry *e*_1_ of *L*_1_ has position element *i*_1_ and “ends” at position *q*_1_ (meaning that *q*_1_ + 1 is the position element of the next entry in *L*_1_, or that *e*_1_ is the last element of *L*_1_ and *q*_1_ is the length of the reference); similarly, assume that entry *e*_2_ of *L*_2_ has position element *i*_2_ and “ends” at position *q*_2_. The intersection of these two entries will be non-empty if and only if *q*_1_ ≥ *i*_2_ and *q*_2_ ≥ *i*_1_. If this is the case, we create an entry *e* for the new genome list *L*_*n*_ corresponding to the intersection segment of *e*_1_ and *e*_2_, which will have starting position max(*i*_1_, *i*_2_) and end position min(*q*_1_, *q*_2_). *L*_*n*_ will contain all such entries resulting from non-empty intersections of entries of *L*_1_ with entries of *L*_2_. See Figure 5A for a graphical representation.

For example, if we assume our usual reference of 20 “A” nucleotides, and consider child genome lists

$$L_{1}=\left\{[N,1,,],\left[R,6,c_{1},\right],\left[T,20,c_{1},\right]\right\}$$

and

$$L_{2}=\left\{[N,1,,],\left[R,4,c_{2},\right]\right\};$$

then we need to consider four intersection fragments:

- The first one from positions 1 to 3 where both lists are of type **N**.
- The second one from position 4 to 5 where *b*_1_ is of type **N** and *b*_2_ is **R**.
- The third one from position 6 to 19 where both children nodes are of type **R**.
- The fourth one at position 20 where *b*_1_ is **T** and *b*_2_ is **R**.

Calculations for each intersection fragment are performed separately, similarly to how calculations for each site in the Felsenstein pruning algorithm are performed independently. We describe this process here considering a general non-empty intersection between en entry *e*_1_ of *L*_1_ and an entry *e*_2_ of *L*_2_ - the whole genome list *L*_*n*_ is generated by repeating this process in order of genome position for each non-empty intersection and concatenating the results in *L*_*n*_. For simplicity, we assume that *e*_1_ = [*τ*_1_, *i*_1_, *c*_1_, *v*_1_] and *e*_2_ = [*τ*_2_, *i*_2_, *c*_2_, *v*_2_], that *i* = max(*i*_1_, *i*_2_), and that the intersection fragment between *e*_1_ and *e*_2_ consists of *λ* nucleotides, that is *λ* = min(*q*_1_, *q*_2_) + 1 − *i*; in case *τ*_1_ = **O** and other similar cases then we have necessarily *λ* = 1. Our aim is to calculate the corresponding entry *e* = [*τ*, *i*, *l*, *v*], which refers to the partial likelihoods for the intersection fragment of *λ* nucleotides starting at position *i* for the internal node *n*; this entry will then be added to genome list *L*_*n*_. We also describe how we update the total likelihood parameter *K* for *n*. Graphical examples of the cases below are given in Figure 5.

- The first case is when at least one of *τ*_1_ and *τ*_2_ is **N** (Figure 5B, C). In this case, at least one of the two children nodes provides no information regarding the *λ* genome positions being considered. Since one child node contributes no information, we only need to pass the genome list entry information of the other child to *n*, while updating its branch length element *l*. The reason why we update this element is that the information on the genome list entry, whether it is in the form of a likelihood vector or an observed nucleotide, now does not refer to node *n*, but to its child node or some further descendant; so, to correctly and efficiently calculate likelihoods we need to keep track of the branch length distances separating partial likelihoods. As an example, in the case *τ*_1_ = **N** we have *e* = [*τ*_2_, *i*, *c*_2_ + *l*_2_, *v*_2_]. Note however that if *τ*_2_ = **N** then we don’t need to keep track of the branch length element of *e* (Figure 5B), and if *τ*_2_ ≠ **O** the partial likelihood vector element of *e* is also unnecessary (Figure 5C).
- The second scenario is the case when *e*_1_ and *e*_2_ are of the same type: *τ*_1_ = *τ*_2_ ∈ {**R**,**A**,**C**,**G**,**T**} (see also Figure 5D). In this case, the two children nodes of *n* support the same nucleotide with negligible uncertainty. Since the evolutionary distances separating the nodes of the tree are assumed to be short, and therefore any mutational history involving a different nucleotide at the parent node would not be parsimonious and would have considerably lower likelihood, then we define *e* to also have the same type *τ* = *τ*_1_ = *τ*_2_. The branch length entry of *e* will be *l* = 0 since type *τ* is considered observed at node *n*, and no partial likelihood vector *v* is required, resulting in *e* = [*τ*, *i*, 0,]. In this case, we also add a contribution to the total likelihood *K* corresponding to the probability of the mutational history with no events; for example, if *τ* = **A**, the log-probability that no mutation event happened along the evolutionary distance *l*_1_ + *l*_2_ + *c*_1_ + *c*_2_ is approximated (see also section 5.3.1) as log(1 + (*l*_1_ + *l*_2_ + *c*_1_ + *c*_2_)*q*_*AA*_) ≈ (*l*_1_ + *l*_2_ + *c*_1_ + *c*_2_)*q*_*AA*_. The same approach is taken for *τ* equal to **C**, **G** or **T**. Notice that this step does not require the computationally demanding calculation of logarithms. The scenario *τ* = **R** works similarly, except that this time we have to add to *K* the log-probability contribution over all *λ* sites of the considered fragment. This is done efficiently by pre-computing the total substitution rate for any prefix stretch of the reference genome $t(i)=\sum_{j=1}^{i}q_{r_{j}r_{j}}$ (with *t*(0) = 0 by definition), where *r*_*j*_ is the nucleotide at position *j* of the reference genome. Then, the total substitution rate for a stretch of *λ* sites from position *i* is *t*(*i* + *λ* − 1) − *t*(*i* − 1) and the approximate log-probability contribution to *K* for the whole stretch of sites is calculated in constant time as (*l*_1_ + *l*_2_ + *c*_1_ + *c*_2_)(*t*(*i* + *λ* − 1) − *t*(*i* − 1)). This step is the key for reducing the number of calculations required at each node from the order of genome size to the order of the number of differences of any lineage with respect to the reference.
- The next case considered is when *τ*_1_ ≠ *τ*_2_ and both *τ*_1_, *τ*_2_ ∈ {**R**,**A**,**C**,**G**,**T**} (Figure 5E). In this case, the two children nodes of *n* support two different nucleotides and so we expect that the two likelihoods corresponding to these two nucleotides for *n* will have similar orders of magnitude, and will typically be larger than the likelihoods of the two nucleotides not supported by any child node of *n*. For these reasons, we set *e* to be of type *τ* = **O**. Also note that due to at least one of the two child nodes not being of type **R** or **N**, we necessarily have *λ* = 1. We can therefore assume for simplicity that *τ*_1_ and *τ*_2_ represent individual nucleotides (that is, if for example *τ*_1_ = **R**, then we can equivalently consider *τ*_1_ as the reference nucleotide at the considered position). We calculate a vector of partial likelihoods *v* since, in the vast majority of cases, the partial likelihoods at this site and node will not be extremely concentrated in one nucleotide. We approximate the relative partial likelihoods at *n* (the entries of *v*) as $p_{i}^{n}(X)\approx \left(\delta_{X\tau_{1}}+q_{X\tau_{1}}\left(l_{1}+c_{1}\right)\right)\left(\delta_{X\tau_{2}}+q_{X\tau_{2}}\left(l_{2}+c_{2}\right)\right)$ following section 5.3.1; here *δ*_*Xτ*_ is the Kronecker delta. We then normalize the vector *v* and add the logarithm of the normalization factor to *K*. Since we have calculated *v* at node *n*, we set *l* = 0, leading finally to entry *e* = [**O**, *i*, 0, *v*].
- The last case is when *τ*_1_ = **O** or *τ*_2_ = **O**. In this case, at least one of the child nodes of *n* has likelihoods not concentrated at a single nucleotide (that is, is of type **O**), and so the same might be true for *n*. To deal with this possibility, we first calculate the vector *v* of partial likelihoods for *n*, and then decide the type *τ* depending on if *v* is concentrated at one nucleotide or not. Here we show as an example likelihood calculation of the most complex case *τ*_1_ = *τ*_2_ = **O**. Again following section 5.3.1, we approximate the partial likelihoods as $v(X)=p_{i}^{n}(X)\approx \left(\sum_{X_{1}}\left(\delta_{XX_{1}}+q_{XX_{1}}\left(l_{1}+c_{1}\right)\right)v_{1}\left(X_{1}\right)\right)\left(\sum_{X_{2}}\left(\delta_{XX_{2}}+q_{XX_{2}}\left(l_{2}+c_{2}\right)\right)v_{2}\left(X_{2}\right)\right)$ where *v*(*X*) is the entry of *v* corresponding to nucleotide *X*. We then again normalize *v* and add the logarithm of the normalization factor to *K*. If only one entry of *v* has a value above the threshold *ϵ*, then the corresponding nucleotide is the only one likely at *n* and site *i*, and so we set *τ* to this nucleotide (if this nucleotide is the reference nucleotide at site *i*, we set *τ* = **R**); Since entries of type **O** are the most computationally demanding, this helps us reduce overall computational demand. As before, we set *l* = 0, leading finally to entry *e* = [*τ*, *i*, 0, *v*], where *v* might be absent in case *τ* ≠**O**.

These calculations are iterated over all intersection fragments, which together represent a partition of all genome positions. Entries of genome list *L*_*n*_ are included in order based on position element *i*. Then, to reduce memory demand and the time demand of the algorithms using genome list *L*_*n*_, if two consecutive entries of *L*_*n*_ are of type **R** and have the same branch length, we merge them into a single entry of type **R**.

The computational demand of this approach is linear in the total number of entries of all the genome lists in the tree. In fact, the maximum computational demand for creating a genome list entry is a constant, no matter the number of sites represented by the entry. This means that, rather than depending on genome size, the computational demand of this approach will typically be dominated by the average number of differences with respect to the reference in a sample. This approach is also easy and efficient to generalize to more complex models (codon models or models with context dependencies, for example) and is not affected by limitations of matrix exponentiation, such as possible numerical instability with non-stationary non-reversible substitution models and computational complexity for large state spaces [1, 12].

### 5.4 Other partial likelihoods

So far we have discussed partial likelihoods of the form discussed in Equation 1. Normally these likelihoods are sufficient for phylogenetic inference. However, when using a non-stationary model, additional types of likelihoods are useful, as shown in [4]. Since the use of non-stationary models is one of our main goals (due to non-reversibility of SARS-CoV-2 evolution [14]), we follow this same approach here, adapted however to our concise likelihood representation.

Each internal node *n* of our binary tree *ϕ*, with the exception of the root, is connected to three other nodes: two children *b*_1_ (the left child) and *b*_2_ (the right child) and the parent node *P*. The partial likelihoods of the previous section, $p_{i}^{n}(X)$ (which we will refer to here as “lower likelihood”), can be considered as the likelihood of the data “arriving to *n*” from *b*_1_ and *b*_2_, that is the likelihood considering the data of all descendants of *b*_1_ and *b*_2_.

In many circumstances, however, for example when we want to evaluate the likelihood score of adding a new sample to the tree as a descendant of a node *n* (discussed in section 5.5), or the likelihood score of removing a subtree and re-grafting it as a descendant of *n* (section 5.6), we need to consider all of the information in the tree and alignment. To do so efficiently it is convenient to have available, at a node *n*, pre-computed likelihoods that account for all of the data. These “overall likelihoods” are:

(4) $$p_{i}^{n↑\to \leftarrow }(X)=p\left(A_{i},X|M,\phi \right)$$

where *A*_*i*_ like before is all the data in the alignment at site *i*, and *M* is the sequence evolution model; here we use arrow ↑ to represent the fact that we consider the data “arriving” at *n* from its parent node, and similarly arrows → and ← referring to the right and left child nodes of *n*, when these exist. These overall likelihoods $p_{i}^{n↑\to \leftarrow }(X)$ can be approximately calculated and concisely represented similarly to lower likelihoods; again, we only keep track of relative likelihoods, so in practice we only record normalized values ${\tilde{p}}_{i}^{n↑\to \leftarrow }(X)$ corresponding to the posterior probabilities nucleotides at node *n* and site *i*, that is, they represent the ancestral state reconstructions [62].

In addition to calculating overall likelihoods genome lists for each node of the tree (either internal or terminal), we also calculate them for branch mid-points at all non-zero length branches; these lists will help us efficiently and accurately perform tree exploration in the following sections.

To calculate overall likelihoods for the root node we need to consider the root frequencies of the nucleotides. The overall likelihoods at the root are the lower likelihoods multiplied by the root nucleotide frequencies: $p_{i}^{n↑\to \leftarrow }(X)=\pi (X)p_{i}^{n}(X)$, where *π*(*X*) is the root frequency of nucleotide *X*. Overall likelihood genome lists can similarly be obtained from lower likelihood genome lists.

Overall likelihood genome lists at non-root nodes of the tree are instead less straightforward to calculate, and to do it efficiently, we define and keep track of two additional sets of likelihoods (corresponding to two additional genome lists) at each node of the tree. The “upper-left” likelihood $p_{i}^{n↑\leftarrow }(X)$ is defined as the likelihood of the data that is “passed on” to *n* from its parent node *P* and its left child *b*_1_. To formally define this likelihood, we call $A_{i}^{n↑}$ all the data in the alignment column *A*_*i*_ that does not represent observations for any descendant of *n*, so containing all data in *A*_*i*_ that is not found in $A_{i}^{n}$. The upper-left likelihood is defined as

(5) $$p_{i}^{n↑\leftarrow }(X)=p\left(A_{i}^{b_{1}},A_{i}^{n↑},X|M,\phi \right),$$

while similarly the upper-right likelihood is defined as

(6) $$p_{i}^{n↑\to }(X)=p\left(A_{i}^{b_{2}},A_{i}^{n↑},X|M,\phi \right).$$

For the root node, given the fact that it does not possess a parent node, its upper-left (respectively, upper-right) likelihood is calculated combining the lower likelihoods of its left child $p\left(A_{i}^{b_{1}}|X,M,\phi \right)$ (respectively, right child $p\left(A_{i}^{b_{2}}|X,M,\phi \right)$) with the root nucleotide frequencies, as done for the overall likelihoods of the root. For all other nodes, instead, we need to combine likelihood vectors using a very similar approach to the algorithm in section 5.3.2. If *n* is a left (right) child of *P*, to calculate $p_{i}^{n↑\leftarrow }(X)$, we need to combine the upper-right (upper-left) likelihoods of *P* with the lower likelihoods of *b*_1_, and similarly for $p_{i}^{n↑\to }(X)$. Finally, to calculate the overall likelihoods at *n* we can use different combinations, for example we can combine the upper-right likelihoods at *n* with the lower likelihoods at *b*_1_.

In addition to calculating overall likelihood genome lists at internal nodes of the tree, we also calculate them at terminal nodes of the tree (corresponding to samples) and at some mid-branch nodes (nodes that we add in the middle of branches that have length beyond a certain threshold). We create these additional overall likelihood genome lists so to also allow efficient placement of new sample near samples already in the tree and as descendants of mid-branch nodes. if a terminal node is the left child of its parent, then its overall likelihood genome list is calculated by combining its lower likelihood with the upper-right likelihood genome list of its parent; similarly if the node is a right child. For mid-branch nodes, again, if the node at the lower end of the branch is the left child of its parent, then we combine the upper-right likelihood genome list of the parent with the lower likelihood of the child; similarly if the node at the lower end of the branch is a right child.

### 5.5 Efficient phylogenetic placement using maximum likelihood

Phylogenetic placement can be described as the task of adding a new sequence onto an existing phylogenetic tree (see e.g., [32]). This can be useful in many applications, for example in identifying the origin of DNA fragments given a set of reference species [32], in identifying the source cases within an epidemic [56], or in online phylogenetic inference (the gradual update of a phylogenetic tree as new sequences are progressively added to a global dataset) [35]. Here we describe our efficient implementation of maximum likelihood phylogenetic placement within MAPLE using the likelihood genome lists presented in Sections 5.2 and 5.4. We use phylogenetic placement specifically within the context of “stepwise addition” (SA, [53]), that is, to construct an initial phylogenetic tree by starting from a tree containing only one sample and iteratively expanding the tree by placing new samples on it one at the time. We describe in Section 5.6 how this is initial tree is improved in the second part of our approach.

For each new sample we want to place on a current tree, first we traverse the tree looking for the most promising region for placement (section 5.5.1); then we search in detail the point on the tree at which to attach the new sample and the length of the new branch that this adds to the tree (section 5.5.2); finally, we update the genome lists in the tree (section 5.5.3), unless the new sample is identical to (or less informative than) a sample already in the tree, in which case the new sample is put aside and is added to the tree only at the end of our approach (section 5.5.4). As we proceed adding samples to the initial tree, we also update estimates of the sequence evolution model (section 5.5.5), which is used both to improve the placement of the following samples, as well as for the following step in MAPLE, namely the search for topological improvements (section 5.6).

In future developments, this algorithm for phylogenetic placement could be used to efficiently perform phylogenetic updates as new sequences become available during a pandemic, or to infer the origin of new cases given a reference phylogeny.

#### 5.5.1 Finding the initial phylogenetic neighborhood for sample placement

We assume that we are given a tree *ϕ* containing only some of the samples, and that we are given one additional sample *s* to add to *ϕ*. Here, we discuss the task of finding the region of *ϕ* where the best placement of *s* is located.

To do this, we traverse the tree starting from the root node, looking for the area of the tree where the placement of *s* would give us the best likelihood score. We typically do not traverse the whole tree, but instead traverse only a small portion of the internal, terminal, and mid-branch nodes of *ϕ*, stopping traversing into subtrees if the placement at their root looks unpromising (Figure 2). For each node *n* we traverse, we use its overall likelihood genome list (representing the relative likelihoods ${\tilde{p}}_{i}^{n↑\to \leftarrow }(X)$) and combine it with the lower likelihood genome list of *s* (which represents the ${\tilde{p}}_{i}^{s}(X)$ relative likelihoods) to obtain the placement score of *s* at *n*; this is done using a simplified version of the algorithm of section 5.3.2, where the simplification comes from the facts that:

- Node *n* is assumed to be ancestral, or, in other words, that *l*_1_ = 0.
- We don’t need to calculate a genome list resulting from the merging, but only the total likelihood *K* contribution from the merging which constitutes the likelihood cost of the placement.

At this stage we use 1/*L* as default value for *l*_2_, where *L* is genome size, but the user of MAPLE can modify this value; our default corresponds to approximately one expected substitution on the new placement branch of *s*. We discuss in section 5.5.2 how the value of *l*_2_ is actually optimized before the placement is concluded.

Using this procedure, we first calculate the placement score at the root node, and then we move to its children nodes and mid-branch nodes, and calculate their placement scores. As we traverse the tree, we keep track of the best placement score found so far for *s*, which we call *B*_*s*_. If, while traversing the tree, the placement score at an internal node *n* worsens substantially (by default by at least 1 log-likelihood unit) at least a certain number of times (by default two times) moving from the direct ancestors of *n* to *n*, then we do not traverse the tree further downward in the subtree of the descendants of *n*, unless the current placement score at *n* is not worse than *B*_*s*_ by at least a certain threshold (by default 24 log-likelihood units).

We do not attempt placement at nodes with a branch length of 0 above them (these nodes are used to represent a polytomy, and we don’t calculate their overall likelihood genome list since it would coincide with the one in their parent node). When the tree traversal is concluded, we retrieve the node (or mid-branch point) where the best placement score has been recorded. During tree traversal, the rooting of the tree can affect the performance of the placement algorithm, although only slightly.

#### 5.5.2 Zooming in on the phylogenetic neighborhood to finalize sample placement

Once we have identified the node (or mid-branch point) in the phylogeny with the best placement likelihood score, we need to identify exactly the point of the phylogeney near this node (or mid-branch point) where the new branch should be attached to the tree, and we need to define the length of this branch. Here we describe how these choices are made based on maximum likelihood.

If the best placement score was found at a mid-branch point, we only consider different possible placements along this branch. Assuming that the preliminary placement is on a branch with length *l*, the exact point of the preliminary placement will be at height *l*/2 along this branch. First, we try to change this height to *l*/4, and if this leads to a placement likelihood improvement, we further attempt height *l*/8, and so on, until we reach below a certain minimum height (by default 1/10*L*). if the likelihood at height *l*/4 is worse than at *l*/2, then we also attempt at moving the placement upward to height 3*l*/4, and if this results in a placement likelihood improvement, we move further up to 7*l*/8, and so on, until a certain maximum height is reached (by default *l* − 1/10*L*). Every time we attempt placement at a new height we need to calculate a new overall likelihood genome list for the existing phylogeny at the new height; this can be done efficiently using the existing genome lists and the nodes above and below the considered branch. Then, we optimize the length of the new branch added to the tree, by similarly attempting at halving or doubling its length up to a minimum (by default 1/10*L*, but a length of exactly 0 is also attempted) or maximum (by default 10/*L*) value.

If the best preliminary placement score is instead at a node *n*, we try to place the sample above *n* (if the branch above *n* has length *l*, we attempt heights < *l*/2 in a procedure similar to above) and below *n*, that is, on any branch leading to any child of *n*. If *n* represents a polytomy (meaning that at least one of the branches directly below *n* has length 0, which is how we represent polytomy within a formally binary tree), we consider all children of *n* to be part of this polytomy. If a child of *n* has a branch above it of length *l*, we only attempt placements at heights > *l*/2 similarly to before. As before, we also optimize the length of the new branch leading to *s* added to the tree.

#### 5.5.3 Updating genome lists

Every time we add a new sample to the tree, we need to update the genome lists (representing relative partial lower likelihoods, total likelihoods, upper left likelihoods, and upper right likelihoods) for the nodes of the tree. Here we describe how this can be done efficiently by only traversing a small portion of the tree each after each new sample placement.

We start a tree traversal from the location of the placement, and update genome lists for the node of the placement and the nodes just above and below it. These updated genome lists are then “passed on” to neighbouring nodes, following the direction in which the tree is traversed. If at any step, the updated likelihoods are identical to the old ones (up to the threshold *ϵ*), making the update unnecessary, the tree traversal in this direction is halted.

For example, assume that a new sample *s* is added to the tree by placing it on the branch above node *n* — this means that now *n* has a new parent node, *P*, of which *n* is the left child and *s* is the right child. We first calculate all the genome lists for *P* using the existing genome lists in the tree and using the lower likelihood genome list for *s*; then we calculate the overall likelihood genome list for *s*; and then we need to update the genome lists of *n* and all of its descendants. To do this, we pass to *n* the upper-right likelihood genome list of *P* and we combine it with the lower likelihood genome list of *n* to calculate the new overall likelihood genome list for *n*. We similarly update the upper-right and upper-left likelihood genome lists of *n*. If the overall likelihood genome list of *n* has not changed in this process, no further updates are performed for the genome lists of the descendants of *n*; otherwise, we pass the new upper-right likelihood genome list of *n* to its left child, and its upper-left likelihood genome list to its right child, and repeat this process for both children. After the update of the genome lists of the descendants of *n* is completed, we proceed similarly in updating the likelihoods of the nodes that are not descendants of the new node *P*.

#### 5.5.4 Dealing with nearly identical sequences

Here we describe an approach that we use to reduce the complexity of the phylogenetic tree: we remove from the tree samples that are identical or less informative than other samples already in the tree.

When placing a sample *s*_1_, if we find that its sequence is identical to another one associated with sample *s*_2_ already in the tree, instead of adding *s*_1_ as its own independent tip to the existing tree, we add it to a specific list of samples identical to *s*_2_. This is because a maximum likelihood placement of *s*_1_ is the one that places *s*_1_ and *s*_2_ exactly at the same spot of the tree, with the two samples separated only by branches of length 0. This is a common approach in maximum likelihood phylogenetics, where only one representative for each set of identical sequences is considered during phylogenetic inference. Then, after the phylogenetic tree relating the representatives is estimated, all samples previously excluded are added to the tree at the same spot as their corresponding representatives to produce the final tree (see e.g. [38]). We take the same approach here.

However, we go one step further, and not only retain only one representative for each set of identical sequences, but also remove sequences that are less informative than others already added to the tree. What we mean by *s*_2_ being less informative than *s*_1_ is that *s*_2_ and *s*_1_ coincide everywhere in their sequence except for positions where *s*_1_ is strictly less ambiguous than *s*_2_ and not in contradiction with it; for example, *s*_2_ might have a “N” character at some position where *s*_1_ has a nucleotide letter or any IUPAC ambiguity code. Another example is when *s*_2_ has ambiguity character “Y” (meaning “C” or “T”); in this case *s*_1_ is more or equally informative than *s*_2_ if it has a “C”, “T”, or “Y” entry at this position. Our definition of informativeness is further described and discussed in section S3, where we also give proofs of the correctness of our way of dealing with these sequences in a maximum likelihood framework. As before, during placement of *s*_2_, if we visit a sample *s*_1_ that has an equally or more informative sequence than *s*_2_, we add *s*_2_ to the specific list of sequences that *s*_1_ represents, halt the placement search for *s*_2_, and do not extend the tree to include *s*_2_. Then, at the end of the tree inference procedure, we add *s*_2_ back to the tree at the same place as *s*_1_.

This approach of dealing with less informative sequences is not only useful here, but is applicable more generally to most phylogenetic inference frameworks, and we propose it as a more efficient extension to the typical approach for dealing with identical sequences.

In order to take full advantage of this procedure, before placing samples on the tree one at the time, we sort them based on their number of ambiguous characters and on the number of differences with respect to the reference. This increases the number of times in which a more informative sequence is placed on the tree before a less informative one, so that we can remove more samples from the phylogenetic inference process and reduce overall computational demand. A more thorough comparison of each pair of samples would remove more sequences, but to be performed would require quadratic time in the number of samples.

#### 5.5.5 Estimating substitution rates

Substitution models are an essential component of maximum likelihood phylogenetic inference (see section 5.3), and we have described how we use a substitution rate matrix *Q* to calculate phylogenetic likelihoods in section 5.3.2. Here we describe how we estimate *Q* during the estimation of the initial phylogeny. The same matrix *Q* is then also used for the final topological improvements described in section 5.6.

We have currently implemented three nucleotide substitution models in our software, the JC69 [27], GTR [54] and UNREST [61] models. In the case of UNREST and GTR, we use as default initial values for the substitution rate matrix SARS-CoV-2 mutation rate estimates from [14], and we use as root nucleotide frequencies the nucleotide frequencies of the reference genome. In the case of GTR and UNREST, we update the substitution rates as more sequences are added to the tree (by default every 40 sequences). For each sequence we add, we record the number of substitutions of each type observed on the branch leading to the new sample; we only consider positions where the total likelihood genome list entry at the node of placement is one of the types **R**, **A**, **C**, **G**, and **T**; where the same is the case for the partial likelihood vector of the new sample; and where these two types differ from each other. As an approximation, we ignore all other possible substitution events (for example those that might have happened as with a new placement an entry of type **O** at a tree node is connected to an entry of type **A** in the placed sample at a given site) which typically represent a minority of cases, and which are harder to label as substitutions. The total number of mutations of each type observed so far (plus initial pseudocounts) are used to update the substitution rates. For UNREST, mutation counts from **A** to **C** are used for the rate *q*_*AC*_, and so on. For the reversible model GTR, mutation counts from **A** to **C** and from **C** to **A** are used for the rate *q*_*AC*_, and so on. Each substitution rate is updated by setting it to the ratio of the new the number of mutations of the same type observed, over the root frequency (the frequency in the reference genome) of the source nucleotide of the considered substitution. Diagonal elements of the substitution rate matrix are defined so that rows of the matrix sum up to 0. The matrix is normalized so that the expected substitution rate is 1. The updated rates are then used to determine placement probabilities for new leaves. After the whole phylogenetic placement is concluded, and all samples have been added to the tree, the substitution model is kept constant for the next inference stage comprising topological improvements (section 5.6).

### 5.6 Tree topology improvement

Section 5.5 described how we build an initial tree by iteratively placing one sample at the time, and how we estimate a substitution rate matrix *Q*. In this section, we describe the second stage of the phylogenetic inference in MAPLE, where we improve the initial tree by modifying its topology and branch lengths.

Maximum likelihood phylogenetic inference methods usually employ topology-changing moves to a current tree in order to explore the phylogenetic tree space and attempt at finding tree topologies with highest likelihood. Typical tree topology change proposals are for example the nearest neighbour interchange (NNI, a short-distance topology change proposal) scheme and subtree pruning and regrafting (SPR, a long-range topology change proposal) scheme [53]. Here, we propose an efficient implementation of the SPR scheme for the scenario considered. The idea behind this approach is to re-adapt the methods for phylogenetic placement described in section 5.5 in order to re-place nodes of the phylogeny (either internal nodes or terminal ones) by severing them from the tree and proposing their placement elsewhere.

#### 5.6.1 Tree traversal loop for SPR initialization

Our approach for improving the initial tree unfolds in a number of tree traversal loops (the default number of loops is 3). At each loop, we traverse the tree from the root to the tips in preorder (first the parent node, then the left child and all its descendants, and finally the right child and all its descendants). At each non-root node *n* we perform an SPR search procedure (described in detail in section 5.6.2) in which we sever the subtree rooted at *n* from the current tree and attempt to regraft it somewhere else.

Before beginning the procedure, however, we evaluate the log-likelihood cost of the current placement of *n* and its subtree — this corresponds to the total log-likelihood of the current tree, minus the log-likelihood of the subtree rooted at *n* (but using improper root nucleotide frequencies all equal to 1.0) and the log-likelihood of its complement (the tree obtained by removing from the current tree the subtree rooted at *n*). This difference represents the contribution of the current placement of *n* to the total likelihood of the current tree, and it can be calculated as described in section 5.5.1 without needing to calculate the individual components of the difference, and only using genome lists of node *n* and its parent.

If this cost is below a certain threshold (default: 0.2 log-likelihood units), then the likelihood gain of a potential SPR move would be very limited and we do not initiate the SPR search procedure or any of the following steps. If the current cost is above the threshold, we first attempt at optimizing the length *l*_*n*_ of the branch above *n* at the current placement (branch lengths are coarsely optimized by recursively halving or doubling the initial length, as described in section 5.5.2). Given the best found value of *l*_*n*_, this will define the current placement cost of *n*, and the SPR search will use branch length *l*_*n*_ as a branch placement length for the other nodes of the tree as well. However, if the new current likelihood cost corresponding to *l*_*n*_ is below our threshold, or if it is not but no SPR improvement is found, then the SPR procedure is converted into a simple branch length change move.

#### 5.6.2 SPR search

Likelihood costs for the current placement of a node *n*, and alternative placements, are calculated like the placement likelihood scores in section 5.5.1, with the difference that now the lower partial likelihoods that are being placed are not necessarily those of a sample, but are often those of an internal node.

Similarly to section 5.5.1, we traverse the tree in search of nodes and mid-branch points which would provide the best fit (highest likelihood) for a new placement/re-graft of *n*. However, the SPR search tree traversal is started at *n*, not at the root, and it does not traverse the subtree rooted at *n*. Another difference of our SPR search compared to our initial placement search is that now, when evaluating alternative placements, we have to consider the fact that *n* needs to be first severed from the tree, which can affect the existing partial likelihood genome lists in the tree. For this reason, as we traverse the tree trying to re-place *n*, we also carry over a genome list representing new partial likelihoods at the considered node following the severing of *n* from the tree. In other words, removing the subtree rooted at *n* from the tree can affect ancestral nucleotide probabilities in the remainder of the tree, and we need to take this into account when searching a new attachment node for *n* and its subtree.

For example, assume that *n* and *n*_2_ are child nodes of *P*, and so by severing *n* from the tree, *P* becomes a node with a single child node. Assume also that *P* is the left child of its own parent node *P*_2_, which we traverse first in order to assess it as a possible new placement of *n*. To do this, we cannot use the current total likelihood genome list of *P*_2_, since it has been calculated considering also the data in the subtree rooted at *n*. Instead, we first calculate an alternative total likelihood genome list of *P*_2_, following the potential severing of *n*, by combining the lower likelihood genome list of *n*_2_ (which replaces the lower likelihood genome list of *P*) and the upper-right likelihoods genome list of *P*_2_. We use this alternative total likelihood genome list to evaluate the placement cost of *n* at *P*_2_. We then also calculate an alternative lower likelihood genome list for *P*_2_ by combining the lower likelihood of *n*_2_ with the lower likelihood of the right child of *P*_2_, and pass it on to the parent node of *P*_2_ as we keep traversing the tree. Similarly, we calculate an alternative upper-left likelihood genome list for *P*_2_ and pass it to its right child as we traverse it. At each step of tree traversal we therefore calculate some alternative genome lists, of which we use the total likelihood one for assessing the placement cost of *n*, while the others we pass on as we move further along the tree traversal. All these alternative genome lists do not immediately replace the old ones, but instead the old ones are stored in case no SPR improved re-grafting is found for *n*.

Usually, after a very few steps in this tree traversal, we find that the alternative partial likelihood genome lists coincide with the pre-existing ones (meaning that changes in the tree like the severing of a subtree typically only affect ancestral state probabilities for a small neighborhood near the severed node), or rather that their difference is below our threshold *ϵ*. When this happens, we avoid the calculation and passing on of alternative genome lists that would necessarily coincide with those already in the tree.

Similarly to section 5.5.1, if, while traversing the tree during an SPR search, we reach a point at which the re-placement of *n* is sufficiently unlikely (by default, more than 60 log-likelihood units more unlikely than the best placement found so far) and if the re-placement cost has increased by at least 1 log-likelihood unit a sufficient number of times (by default three times) while traversing the tree in the same direction, then we stop the SPR search in that direction.

Note that some of our heuristics for SPR search are similar to some that have been developed for other phylogenetic packages, and in particular RAxML. For example, our SPR approach is similar to the Lazy Subtree Rearrangement (LSR [49]), with some differences: we don’t optimize three branch lengths at each SPR evaluation, but instead keep constant the sum of the lengths of two of the branches near the re-graft node; also, we don’t define the SPR search radius based on the number of nodes traversed from the original location of the subtree, but instead we only use the difference of the log likelihood score of the proposed SPR moves against the original tree (approach that in itself is similar to the one in [48]). This means that our SPR moves can potentially be more costly than LSR steps, since we could in principle explore a broader region of the tree, for example for subtrees containing extremely uninformative sequences, whose placement can be uncertain; however, at the same time, our log likelihood thresholds means that we usually avoid traversing areas of the tree where the re-placement of a subtree would be very unlikely.

#### 5.6.3 SPR move finalization

If the SPR search finds a better placement point than the current location of *n*, we first sever *n* and its descendants from the tree and update all the genome lists in the rest of the tree accordingly (but again avoiding updating genome lists when the old one and the new are expected to coincide). Then, we place *n* to its new location in the tree and we re-update the genome lists in the tree, starting from *n* and traversing its subtree and the rest of the tree (but again stopping every time that new and old genome lists coincide). Thanks to the stopping criteria for these tree traversals, these genome list updates are usually not very demanding since only a small part of the tree is affected and therefore traversed.

### 5.7 Software implementation

We implemented our methods in a Python3 script available from https://github.com/NicolaDM/MAPLE. One advantage of our simple implementation is that it can be executed with the pypy3 implementation of Python https://www.pypy.org/#!, which makes it substantially faster than when run with python3.

### 5.8 Phylogenetic inference methods used to assess the performance of MAPLE

Here we describe the different phylogenetic methods that we compare to MAPLE in terms of computational demand and accuracy. We considered efficient and popular maximum likelihood phylogenetic methods that are often used to analyse large sequence datasets: IQ-TREE 2 v2.1.3 [39], FastTree 2 v2.1.11 (double precision, no SSE3) [42], and RAxML-NG v1.0.2 [28]. For all these methods we adopt a GTR substitution model [54] unless otherwise specified. Additionally, we compare our approach to the recent parsimony-based method matOptimize [63], which has been specifically developed to address the computational demand of SARS-CoV-2 datasets. Here we focus on method options considered in results presented in the main text; in the Supplementary Material we describe additional, typically slower options (Section S2) and present the results from all options (Section S1).

For all IQ-TREE 2 runs we used options “-quiet” to reduce screen output and “-nt 1” to use only one core per replicate on our cluster. We also used option “-fast”, with which only nearest neighbour interchange (NNI) moves are used.

FastTree 2 was executed with options “-quiet” to limit screen output, “-nosupport” to skip support value computations, and “-nocat” to ignore rate variation. We also used option “-fastest” to reduce the time demand of NNI steps.

RAxML-NG was run with options “–threads 1” to use only one core per replicate on our cluster. We also used option “–blmin 0.000005” to increase the minimum branch length considered and option “–tree pars1” to start the tree search from a parsimony tree.

UShER and matOptimize were run with option “-T 1” to utilize a single thread per replicate, and were run using the vcf input file format (option “-v”). matOptimize was run starting from the initial tree estimate of UShER. Option “-n” was used in matOptimize to avoid the creation of intermediate files.

In order to speed up execution of MAPLE, we use PyPy (v7.3.5 with GCC 7.3.1 20180303 for Python 3.7.10; see https://www.pypy.org/#!). We perform initial phylogenetic placement with 5 allowed failed moves per direction, log-likelihood threshold of 80 units, 2 follow-up topological improvement tree traversals with 3 allowed fails per direction, log-likelihood threshold of 80 while looking for re-placement, and log-likelihood threshold of 0.5 to initialize the re-placement search.

### 5.9 Real SARS-CoV-2 sequence data

For all results in the main text, we ran phylogenetic estimations on SARS-CoV-2 datasets obtained by randomly subsampling without replacement a given number of sequences from the 540,520 whole genomes that were represented both in 31st of March 2021 global unmasked SARS-CoV-2 alignment from GISAID and in the corresponding phylogenetic tree (see https://www.gisaid.org/ [46]). Datasets of different numbers of sequences were randomly subsampled so to recreate scenarios of varying computational complexity; we always consider the whole genome (29981 columns in the multiple sequence alignment). We did not mask sites or filter out sequences from this dataset, since our aims include developing and investigating methods that are reliable enough to help identify outlier sequences and error-prone sites from the alignment (see e.g. [13, 55]). In particular, the focus of the present work is not in itself in reconstructing the evolutionary history and spread of SARS-CoV-2, for which filtering and masking would instead likely be beneficial. We use the consensus of all the sequences in the global GISAID alignment as reference genome for MAPLE and MAPLE format files.

When measuring running time of different methods, we did not consider the cost of creating the input alignment for a given method. For MAPLE, we did not consider the time required for creating the concise input file (MAPLE format, representing input sequences in terms of differences with respect to the reference), which is however negligible; similarly we did not consider the time required for generating VCF files for UShER and matOptimize, or Fasta and phylip format files for the other maximum likelihood methods considered.

### 5.10 Simulated SARS-CoV-2 sequence data

While real data has the advantage of being more realistic, we also use simulated genome data since it allows us to know the true phylogenetic tree (and so to accurately detect phylogenetic inference errors) and since it allows us to control the complexity of the evolutionary model and disentangle which features of the evolutionary process might affect different methods.

To simulate SARS-CoV-2 alignments we used the publicly available 26th of October 2021 global SARS-CoV-2 phylogenetic tree from http://hgdownload.soe.ucsc.edu/goldenPath/wuhCor1/UShER_SARS-CoV-2/ [35] representing the evolutionary relationship of 2,250,054 SARS-CoV-2 genomes and obtained using UShER [56]. We used phastSim v0.0.3 [15] to simulate sequence evolution along this tree according to the non-reversible non-stationary neutral mutation rates estimated in [14] and using the SARS-CoV-2 Wuhan-Hu-1 genome [60] as root sequence.

We used three different scenarios for our simulated datasets:

- In the basic simulation scenario, no rate variation is simulated and the output of phastSim is not modified.
- In the second scenario, with rate variation, we simulated four categories of sites; the four categories have the same frequency, and relative substitution rates of 0.1, 0.5, 1 and 2 respectively.
- In the third scenario, with sequence ambiguity, we aimed at modeling incomplete sequences and ambiguous characters as are regularly observed in SARS-CoV-2 whole-genome sequences and likely due to amplicon dropout and sample mixture and contamination (see e.g. [13, 55]). To do this, we modified the sequence data of the basic simulation scenario to include ambiguous characters. To make this step as realistic as possible, for each simulated sequence, we sample on random sequence from the real dataset and copy-paste from it the stretches of “N” and gap “-” characters into the simulated sequence. Additionally, we count the number of isolated ambiguous characters in the real sequence, and we mask the same number of SNPs (differences with respect to the reference genome) in the simulated sequence by randomly selecting them. If more isolated ambiguous characters are observed in the real sequence than SNPs in the simulated sequence, then we simply mask all SNPs in the simulated sequence.

### 5.11 Comparison of method performance

We measured the computational demand of different approaches in estimating phylogenies by tracking the running time, average memory demand, and maximum memory demand of all methods. All methods were run on our computer cluster at EMBL-EBI in parallel, assigning one thread per replicate per method. Since matOptimize requires an initial run of UShER, the running time of matOptimize is defined as the sum of the time it took to execute UShER followed by matOptimize; the maximum memory demand for matOptimize was defined as the highest of the maximum memory demands of the two methods; as average memory demand of matOptimize we take the maximum between the two average memory demands.

To compare the accuracy of different approaches we used two methods. The first method is to compare the likelihoods of the trees estimated by different approaches. This method is particularly useful in the analysis of real data, for which the correct tree is not know. Trees with higher likelihoods are typically interpreted as representing better estimate, as assumed by the maximum likelihood paradigm. To run our comparison, we first run each considered inference method to estimate a tree. Then, we compare the tree topologies inferred by different methods by computing the likelihoods of these different topologies with the same method, which is IQ-TREE 2 with model and branch length optimization but without topological improvements. This means that while maximum likelihood methods were used to estimate topologies, branch lengths and substitution rates, we only compare here the estimated topologies by re-estimating branch lengths and substitution rates in IQ-TREE 2 given each inferred topology. We do this since parsimony-based approaches don’t provide likelihoods, substitution rate estimates, or branch lengths comparable with maximum likelihood methods, and since the likelihoods computed by different maximum likelihood methods may not be comparable. In simulations with rate variation we evaluate topology likelihoods in IQ-TREE 2 using a GTR+G model with four categories (which is slightly different from the rate variation model used in simulations, but which is available in IQ-TREE 2), while in all other cases we use a GTR model without rate variation.

A second method that we use to measure phylogenetic accuracy on simulated data is to calculate the Robinson-Foulds distance [44] between an inferred tree and the corresponding true simulated tree. This method cannot be used with real data since in that case we don’t know the true phylogenetic tree to compare inferred trees to. When calculating Robinson-Foulds distances we consider the trees as unrooted, and we collapse all branches shorter than a minimum branch length (defined by the minimum branch length considered by each method) so as to represent trees as multifurcating when there is little or no support for local branching order. When comparing multifurcating trees, we consider a multifurcation as the absence of a certain number of bifurcations (see [10]). To further increase the interpretability of the results, instead of comparing the inferred trees to the input trees used for sequence simulations in phastSim, we compared them to the trees of realized mutation events, that is, we collapse all branches of the simulation trees on which no simulated mutation events occurred and which are therefore not inferrable by any method. For efficiency, and to adopt the custom features mentioned above, Robinson-Foulds distance calculations were performed with our own custom implementation of Day’s algorithm [10].

## Supplementary Material

1

## Figures and Tables

**Table 1:** Explanation of main symbols and expressions used in the Methods section.

Expression	Meaning
*ϕ*	Phylogenetic tree
*n*	Generic node of *ϕ*
*L*	Genome length (number of alignment columns)
*i*	Generic genome position 1 ≤ *i* ≤ *L*
*A*	Genetic data (multiple sequence alignment)
*A*_*i*_	Column *i* of alignment *A*
$A_{i}^{n}$	Sub-vector of *A*_*i*_ corresponding to all the descendants of *n*
*M*	Model of sequence evolution
*X*	Generic nucleotide
$p_{i}^{n}(X)$	Partial likelihood at node *n* and position *i* conditional on nucleotide $X:p\left(A_{i}^{n}|X,M,\phi \right)$
${\tilde{p}}_{i}^{n}(X)$	Relative (normalized) likelihood: $p_{i}^{n}(X)/\sum_{D}p_{i}^{n}(D)$
*e*	Generic genome list entry *e* = (*T*, *i*, *l*, *v*)
*τ*	Generic genome list entry type ∈ {**A**,**C**,**G**,**T**,**R**,**N**,**O**}
*l*	Evolutionary distance from the node of calculation of the partial likelihoods represented by the genome list entry
*v*	Generic vector of partials ${\left({\tilde{p}}_{i}^{n}(X)\right)}_{X}$
*ϵ*	Lower threshold of negligibility for relative likelihoods ${\tilde{p}}_{i}^{n}(X)$
*K*	Total likelihood, tracks the likelihood contributions across the genome and the subtree of the considered node
*L*_*n*_	Genome list at node *n*
*t*(*i*)	Cumulative substitution rate up to reference position *i*
*M*	The substitution model
π(*X*)	Root frequency of nucleotide *x*
*Q*	The substitution rate matrix
$q_{X_{1}X_{2}}$	Substitution rate from nucleotide *X*_1_ to *X*_2_
−*q*_*XX*_	Total substitution rate from nucleotide *X*
*r*_*i*_	Nucleotide at position *i* of the reference genome
$A_{i}^{n↑}$	Data at position *i* for the tree except the descendants of *n*
$p_{i}^{n↑\leftarrow }(X)$	Likelihood of the data from left child and parent node
$p_{i}^{n↑\to }(X)$	Likelihood of the data from right child and parent node
$p_{i}^{n↑\to \leftarrow }(X)$	Likelihood for all data
*s*	Generic sample
