# Supplementary material for: Maximum likelihood pandemic-scale phylogenetics
Source: bioRxiv. 2022 Mar 22:2022.03.22.485312. Preprint. [Version 1] doi: 10.1101/2022.03.22.485312 (PMC8963701; doi:10.1101/2022.03.22.485312)
Supplement: 1 [file NIHPP2022.03.22.485312V1-supplement-1.pdf]

bioRxiv preprint doi: https://doi.org/10.1101/2022.03.22.485312; this version posted March 22, 2022. The copyright holder for this preprint (which was not certified by peer review) is the author/funder, who has granted bioRxiv a license to display the preprint in perpetuity. It is made available under aCC-BY 4.0 International license.

Supplement

S1 Supplementary figures

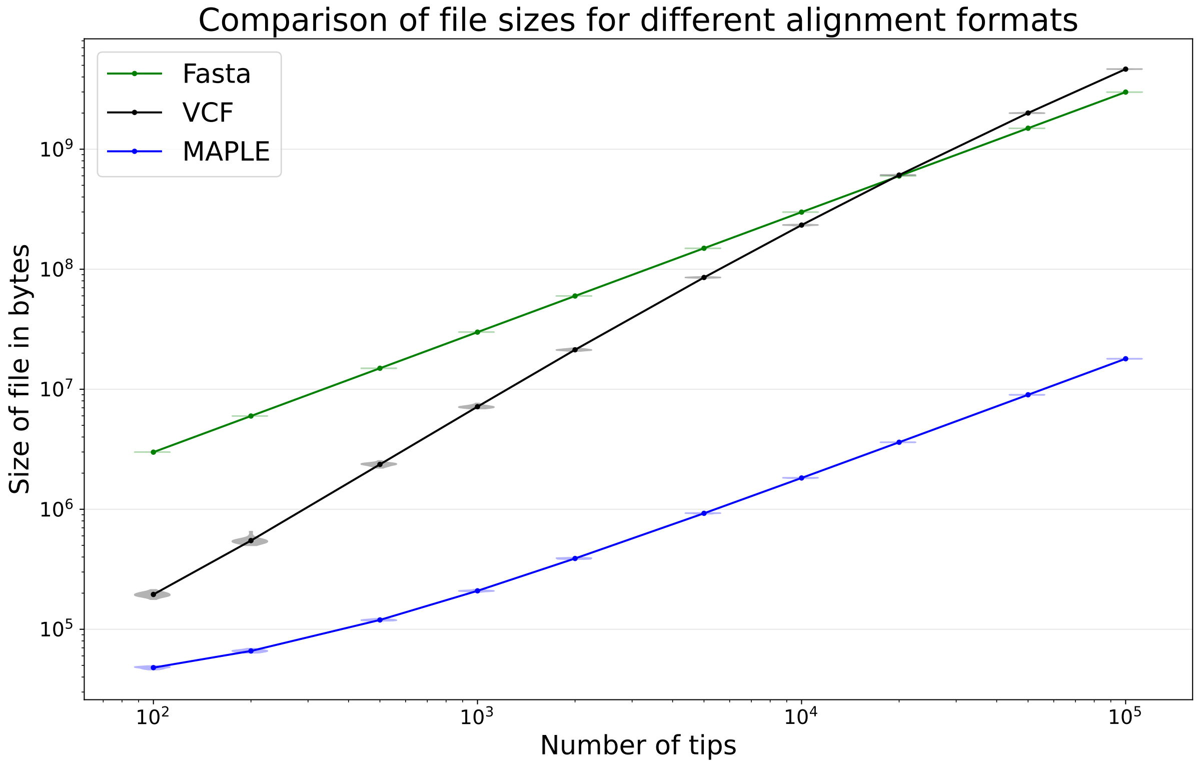

Figure S1: **Comparison of file sizes of SARS-CoV-2 genome alignments using different alignment formats**. On the Y axis on a logarithmic scale we show the sizes of alignment files for each format considered, expressed in bytes. On the X axis is the number of sequences in the dataset considered on a logarithmic scale. Here we consider random subsamples of our real SARS-COV-2 alignment data; see Section 5.9. Violin plots (often variation within one plot is not visible, collapsing the violin plots into horizontal lines) summarize values for 20 replicates, and dots represent their mean.

bioRxiv preprint doi: https://doi.org/10.1101/2022.03.22.485312; this version posted March 22, 2022. The copyright holder for this preprint (which was not certified by peer review) is the author/funder, who has granted bioRxiv a license to display the preprint in perpetuity. It is made available under aCC-BY 4.0 International license.

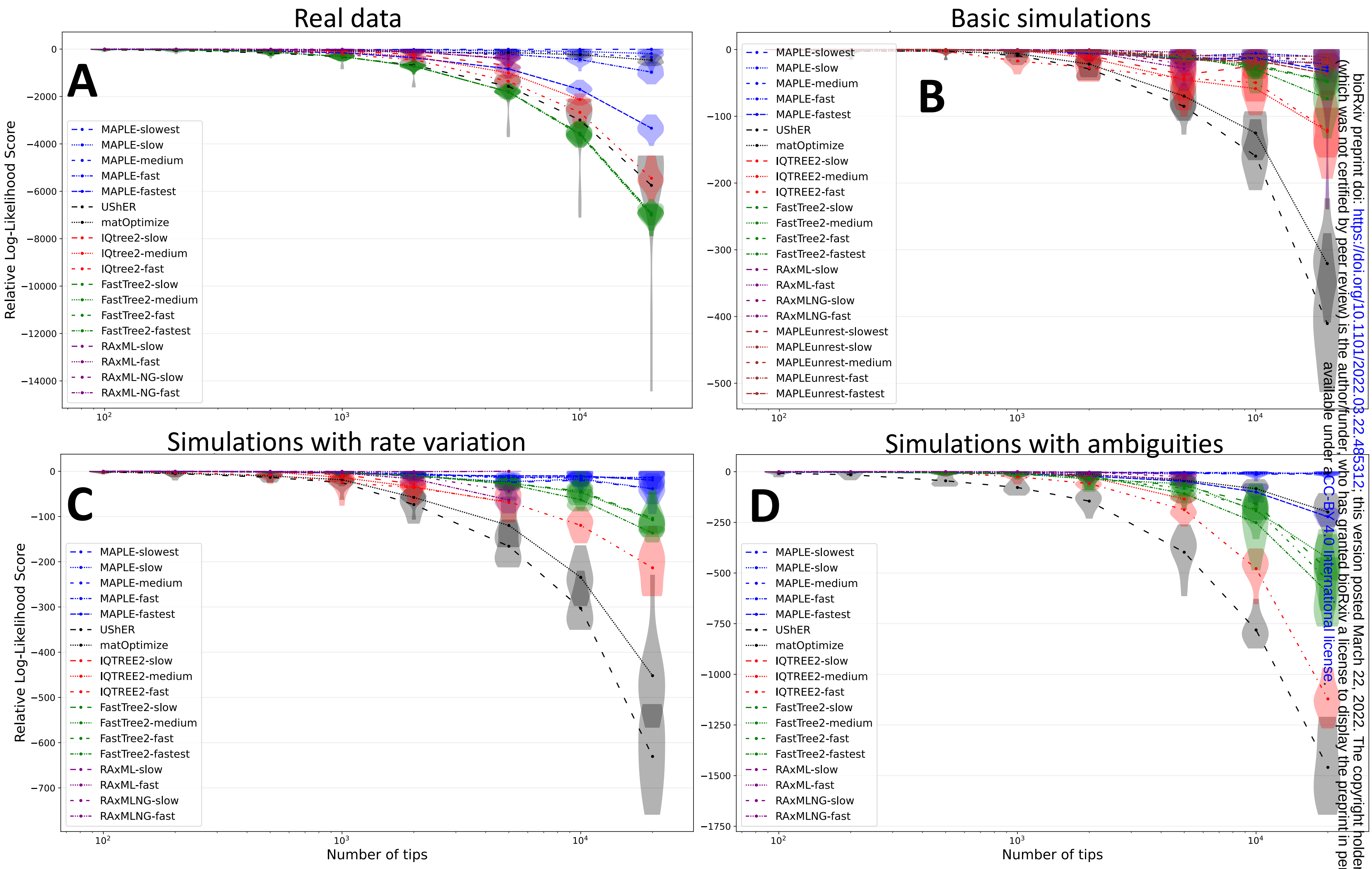

Figure S2: **Comparison of relative likelihood scores of trees inferred by different phylogenetic methods from SARS-CoV-2 genomes**. MAPLE leads to more accurate tree reconstruction (tree topologies with higher likelihoods) both in real data and simulations. On the Y axis we show the relative log-likelihood scores of the tree estimated by each method, as in Figure 3, with higher scores representing more likely tree estimates. On the X axis is the number of sequences in the dataset considered on a logarithmic scale. Different line styles and colors represent different options for each method, as denoted in the legend and Section S2. We ran each method and set of options up to the maximum dataset size that was achievable on our computer cluster due to time and memory limitations. Violin plots summarize values for 20 replicates, and dots represent their mean. **A** Results for subsamples from the real SARS-CoV-2 dataset. **B** Simulated datasets with no rate variation or ambiguity. **C** Results on simulated data with rate variation but no ambiguities. **D** Simulated data with no rate variation but with ambiguities.

bioRxiv preprint doi: https://doi.org/10.1101/2022.03.22.485312; this version posted March 22, 2022. The copyright holder for this preprint (which was not certified by peer review) is the author/funder, who has granted bioRxiv a license to display the preprint in perpetuity. It is made available under aCC-BY 4.0 International license.

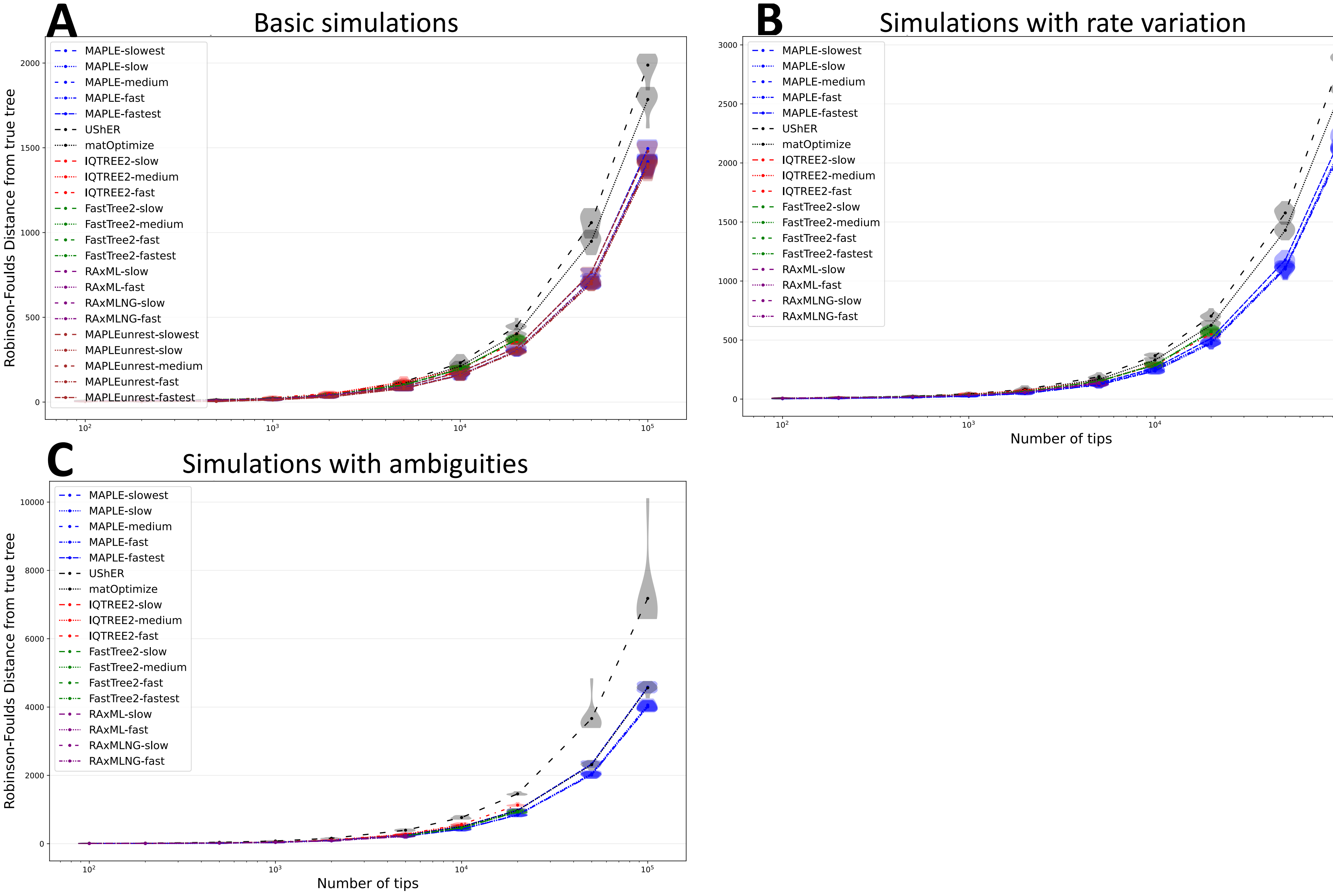

Figure S3: **Comparison of Robinson-Foulds distances of inferred trees from the correct simulated trees**. On the Y axis we show the Robinson-Foulds distances of the tree estimated by each method with respect with the true simulated tree of the corresponding scenario and replicate. We collapsed tree branches of the simulated trees where no mutation event was simulated. Trees were compared as unrooted, and polytomies were compared as such (we collapsed branches of inferred trees with length equal to the minimum allowed length by the corresponding inference method). **A** Results for simulated datasets with no rate variation or ambiguity. **B** Results on simulated data with rate variation but no ambiguities. **C** Simulated data with no rate variation but with ambiguities. Other details are the same as in Figure S2.

bioRxiv preprint doi: https://doi.org/10.1101/2022.03.22.485312; this version posted March 22, 2022. The copyright holder for this preprint (which was not certified by peer review) is the author/funder, who has granted bioRxiv a license to display the preprint in perpetuity. It is made available under aCC-BY 4.0 International license.

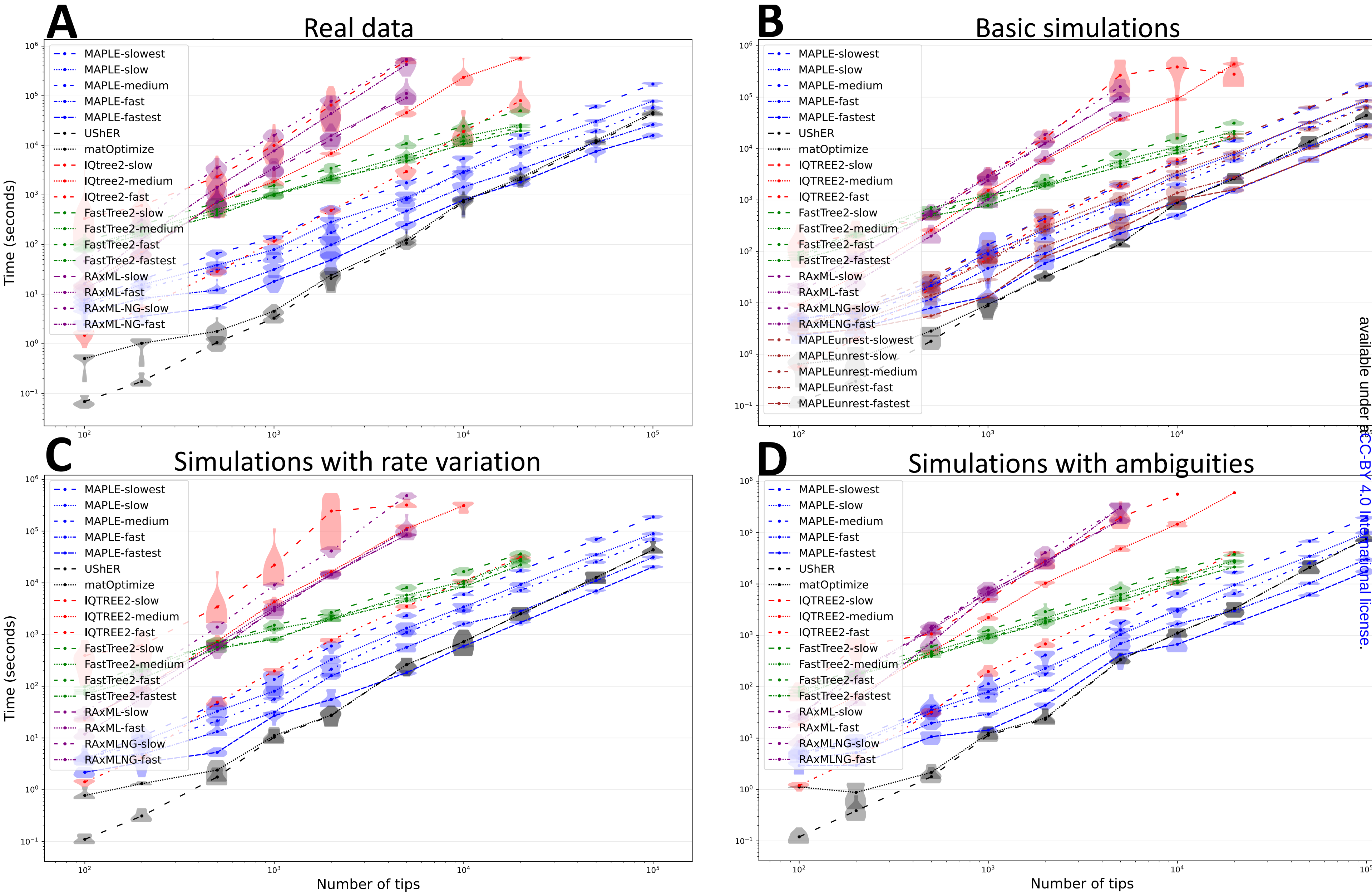

Figure S4: **Comparison of running times of all considered methods and options for phylogenetic inference from SARS-CoV-2 genomes**. On the Y axis on a logarithmic scale we show the number of seconds it takes to run each method. Other details are the same as in Figure S2.

bioRxiv preprint doi: https://doi.org/10.1101/2022.03.22.485312; this version posted March 22, 2022. The copyright holder for this preprint (which was not certified by peer review) is the author/funder, who has granted bioRxiv a license to display the preprint in perpetuity. It is made available under aCC-BY 4.0 International license.

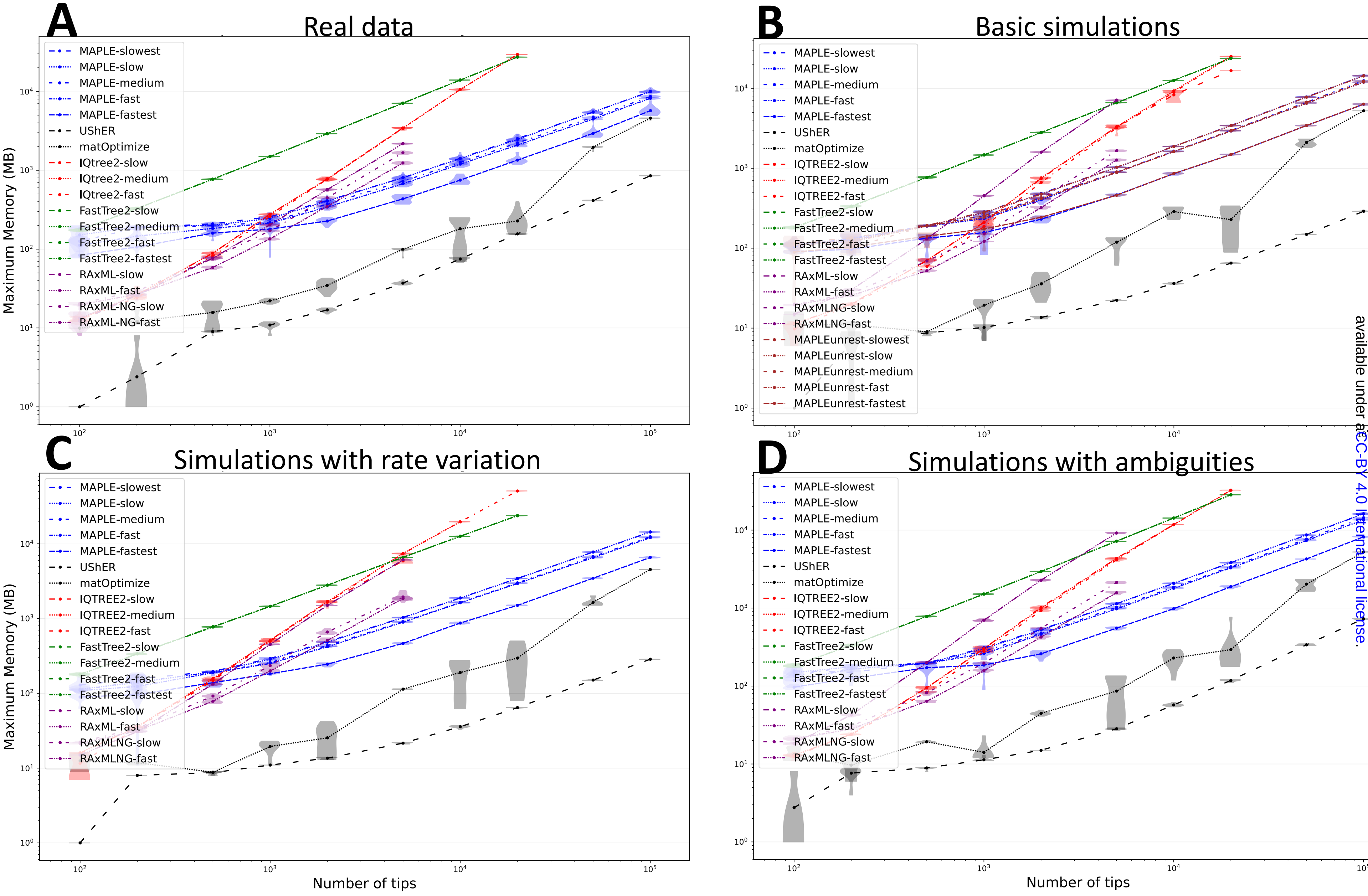

Figure S5: **Comparison of maximum memory demand of all considered methods and options for phylogenetic inference from SARS-CoV-2 genomes**. On the Y axis on a logarithmic scale we show the maximum RAM memory demand in MB required to run each method. Other details are the same as in Figure S2.

bioRxiv preprint doi: https://doi.org/10.1101/2022.03.22.485312; this version posted March 22, 2022. The copyright holder for this preprint (which was not certified by peer review) is the author/funder, who has granted bioRxiv a license to display the preprint in perpetuity. It is made available under aCC-BY 4.0 International license.

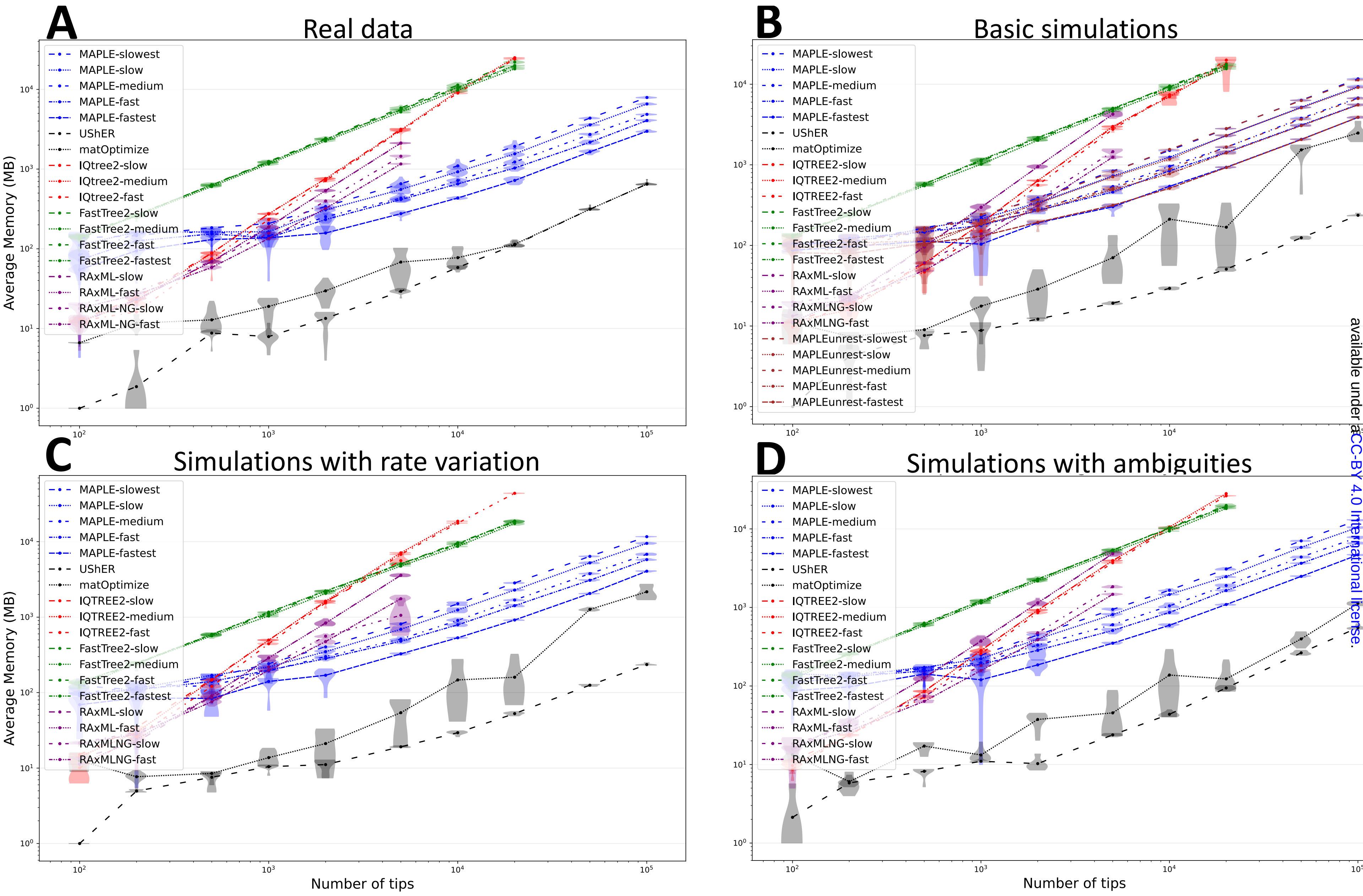

Figure S6: **Comparison of average memory demand of all considered methods and options for phylogenetic inference from SARS-CoV-2 genomes**. On the Y axis on a logarithmic scale we show the mean RAM memory demand in MB required to run each method. Other details are the same as in Figure S2.

bioRxiv preprint doi: https://doi.org/10.1101/2022.03.22.485312; this version posted March 22, 2022. The copyright holder for this preprint (which was not certified by peer review) is the author/funder, who has granted bioRxiv a license to display the preprint in perpetuity. It is made available under aCC-BY 4.0 International license.

S2 Extended comparison with other phylogenetic inference methods

Sometimes specific phylogenetic method options used in can affect the computational performance and accuracy of phylogenetic inference. For this reason, to extend our comparison between phylogenetic methods, we considered a number of options for the different methods considered.

We test the performance of MAPLE using five option settings, from fastest to slowest:

- Fastest: initial phylogenetic placement with 3 allowed failed moves per direction, log-likelihood threshold of 40 units, no follow-up topological improvements. Since we do not perform any SPR moves but just estimate an initial tree, this option is the fastest one considered here.
- Fast: initial phylogenetic placement with 4 allowed failed moves per direction, log-likelihood threshold of 60 units, 1 follow-up topological improvement tree traversal with 2 allowed fails per direction, log-likelihood threshold of 60 while looking for re-placement, and log-likelihood threshold of 1 to initialize the re-placement search. This option does perform SPR moves to try to improve the initial tree, but is still fast since the number of SPR moves attempted is small.
- Medium (also considered in the main text): initial phylogenetic placement with 5 allowed failed moves per direction, log-likelihood threshold of 80 units, 2 follow-up topological improvement tree traversal with 3 allowed fails per direction, log-likelihood threshold of 80 while looking for re-placement, and log-likelihood threshold of 0.5 to initialize the re-placement search.
- Slow: initial phylogenetic placement with 5 allowed failed moves per direction, log-likelihood threshold of 100 units, 3 follow-up topological improvement tree traversal with 4 allowed fails per direction, log-likelihood threshold of 100 while looking for re-placement, and log-likelihood threshold of 0.2 to initialize the re-placement search.
- Slowest: initial phylogenetic placement with 5 allowed failed moves per direction, log-likelihood threshold of 120 units, 5 follow-up topological improvement tree traversal with 6 allowed fails per direction, log-likelihood threshold of 150 while looking for re-placement, and log-likelihood threshold of 0.1 to initialize the re-placement search.

The difference between our slower and our faster options is that the slower ones perform a more in-depth tree search, and so take longer but are also expected to result in trees with higher likelihoods. In some simulation settings, we additionally also run our approach using an UNREST substitution model - unlike other maximum likelihood methods, MAPLE does not use rate matrix exponentiation, which means that we can make numerically stable use of non-reversible models.

For IQ-TREE 2 we considered three different speed settings:

- Fast (also considered in the main text): we used option "-fast", for which only nearest neighbour interchange (NNI) moves are used.
- Medium: we used default options and "-blmin 0.000000005" to allow shorter branch lengths.

bioRxiv preprint doi: https://doi.org/10.1101/2022.03.22.485312; this version posted March 22, 2022. The copyright holder for this preprint (which was not certified by peer review) is the author/funder, who has granted bioRxiv a license to display the preprint in perpetuity. It is made available under aCC-BY 4.0 International license.

- Slow: in addition to "-blmin 0.000000005" we also used options "-nstop 500" to increase the number of unsuccessful iterations before stopping, and "-pers 0.1" to decrease the default perturbation strength.

For FastTree 2 we used four speed settings:

- Fastest (also considered in the main text): we used option "-fastest" to reduce the time demand of NNI steps.
- Fast: default setting.
- Medium: we used default options and "-spr 4" to increase the number of rounds of minimum-evolution SPR moves.
- Slow: in addition to "-spr 4" we also used options "-mlacc 2 -slownni" to make the maximum-likelihood NNIs more exhaustive.

RAxML-NG we considered two speed settings:

- Fast (also considered in the main text): we used option "–blmin 0.000005" to increase the minimum branch length considered and option "–tree pars1" to start the tree search from a parsimony tree.
- Slow: we used option "–blmin 0.000000005 –tree pars3" to decrease the minimum branch length and start the tree search from 3 parsimony trees.

In addition to RAxML-NG, we also ran RAxML v8.2.11 (raxmlHPC) [47] using options "-F" to skip gamma model estimation, and "-c 1 -V" to skip the model of rate variation. We ran RAxML with two speed settings:

- Fast: we used option "-D" to speed up tree search.
- Slow: default settings.

matOptimize and UShER were run either one after the other (as considered in the main text), or, as a faster and less memory intensive alternative, we ran UShER not followed by topological improvements with matOptimize.

S3 Dealing with identical sequences in maximum likelihood framework

When performing maximum likelihood phylogenetic inference, it is typical to discard identical sequences (keeping in the analysis only one representative of a set of identical sequences). This is because in typical analyses at least one maximum likelihood tree will have all identical sequences clustered together (equivalently, separated by 0 branch length), as we discuss below. So, one can keep only one representative for each set of identical sequences, estimate a maximum likelihood tree, and then at the end re-attach the excluded copies to the tree at the same location as their representative. In Bayesian inference, it is also possible, although more challenging, to achieve some computational savings in this scenario [2]. In the following we consider maximum likelihood phylogenetic inference. We will assume that we are using a substitution model without sequencing errors. In these settings, we show that it is possible to extend the approach above and remove more than just identical sequences.

bioRxiv preprint doi: https://doi.org/10.1101/2022.03.22.485312; this version posted March 22, 2022. The copyright holder for this preprint (which was not certified by peer review) is the author/funder, who has granted bioRxiv a license to display the preprint in perpetuity. It is made available under aCC-BY 4.0 International license.

S3.1 Possible issues to consider when removing sequences

Recall the fact that multiple maximum likelihood trees might exist for the same dataset. For example, if we consider the following three sequences, each 2bp long: AA, AC, AN, then the tree ((AC:0,AN:0):l,AA:l) will have, under a reversible substitution model, the same likelihood as the tree ((AA:0,AN:0):l,AC:l). The approach we will describe might introduce biases with respect the selection of one of such trees versus another. If our aim is to estimate any maximum likelihood tree, then this is not an issue. However, if one also aims to represent the uncertainty in maximum likelihood inference, for example as in phylogenetic bootstrap [20], then this limitation has to be considered. Of course, the same is true with the traditional approach of removing strictly identical sequences.

S3.2 Extension of the sequence removal approach

We will represent the fact that two aligned sequences a and b are identical as "$a = b$", and the fact that a is strictly (non-strictly) more informative than b as "$a > b$" ("$a \geq b$"). What we mean by a being "more informative" than b, is that any allele that is possibly present at a position of the genome of the sample of a, could possibly also be present at the same position in b. In the simple case of an alignment made of a single column, we have that A>N, while A=A, but A and C are not comparable (A$\ngeq$C and C$\ngeq$A). For the following, gaps are treated as the same as ambiguous "N" characters. The other relations for other IUPAC ambiguity codes follow similarly, for example, since Y represents "C or T", we have that C>Y, that Y>N, and that Y and A are not comparable. For longer alignments/sequences, we can simply say that for sequences a and b we have $a \geq b$ if and only if $\forall i, a_i \geq b_i$ where a_i is the ith character of sequence a (or more precisely the character of a in alignment column i). Under these definitions, the sequences within an alignment form a partially ordered set.

Our extended strategy is motivated by the following fact:

Lemma S3.1. *If $a \geq b$, then the tree obtained removing b from the alignment, estimating a maximum likelihood tree, and re-adding b to form a 0-branch-length clade with a, is still a maximum likelihood tree.*

Proof. Let's call T_{-b} the maximum likelihood tree obtained without b. The tree obtained by adding b to it will be T_{-b+b}, and we have that T_{-b} and T_{-b+b} have the same likelihood, since one can think of b as a descendants of a with 0 distance from it, and no substitutions from it. Now, let's assume *ad absurdum* that a tree T exists for the whole alignment with a higher likelihood than T_{-b+b}. We can then remove b from T and obtain a tree $T - b$ with the same or higher likelihood than T. This means that $T - b$ has a higher likelihood than T_{-b}, which is absurd since T_{-b} was a maximum likelihood tree, and which proves the lemma. □

Lemma S3.1 can of course be generalised to any number of sequences: if we have $B = \{b_1 \dots b_n\}$ a set of sequences that we remove from the alignment, and $\forall i, b_i \leq a_i$ for some a_i not removed from the alignment, then we can remove all the sequences in B, estimate a tree, and then re-add B to the tree "attaching" each to b_i to the corresponding a_i, and we would still have a maximum likelihood tree - which can be proven by sequentially applying lemma S3.1.

bioRxiv preprint doi: https://doi.org/10.1101/2022.03.22.485312; this version posted March 22, 2022. The copyright holder for this preprint (which was not certified by peer review) is the author/funder, who has granted bioRxiv a license to display the preprint in perpetuity. It is made available under aCC-BY 4.0 International license.

This means that, in order to infer a maximum likelihood tree, we only need to infer a maximum likelihood tree for the maximal sequences (the sequences that have no other sequence more informative than them) in the alignment, where we pick only one representative among identical maximal sequences. While the set of maximal sequences (without duplicates) is unique and well-defined, the way we re-attach removed sequences to create the full maximum likelihood tree is not necessarily unique. This is because, generally, a sequence may have multiple different more informative sequences, and so it might have multiple identically optimal attachment locations, as in the example in Section S3.1; we do not consider this an issue in our current work, but this is something that should be considered carefully when, for example, using this approach when calculating bootstrap values, since systematically clustering together identical sequences when in reality the might be attached to various more informative sequences might lead to overestimating the support for their clustering, both in our generalized approach as well as in the classical approach of removing only identical sequences.

So, in summary, we want to find a partition of the sequences in the alignment, where the sequences in each set of the partition have at least one maximum element (a sequence at least as informative as any other sequence in the set). One maximum from each set can be used as representatives to infer a maximum likelihood tree, and then all the other elements of the sets can be attached to the corresponding maximum. A partition where the number of sets, and therefore the number of maxima, is equal to the number of maximal elements in the alignment (without counting duplicates), will be optimal, meaning that will have the minimum possible number of sets, and should therefore make maximum likelihood inference the fastest. Note that, in this case, the set of maxima and the set of maximal elements (without duplicates) should coincide.

Note that from Lemma S3.1 above also follows the proof of the special case of the standard approach of removing only identical sequences.
